# Phosphorylation of Rab29 at Ser185 regulates its localization and role in the lysosomal stress response in concert with LRRK2

**DOI:** 10.1242/jcs.261003

**Published:** 2023-07-21

**Authors:** Tadayuki Komori, Tomoki Kuwahara, Tetta Fujimoto, Maria Sakurai, Ikuko Koyama-Honda, Mitsunori Fukuda, Takeshi Iwatsubo

**Affiliations:** ^1^ Department of Neuropathology, Graduate School of Medicine, The University of Tokyo, Tokyo 113-0033, Japan; ^2^ Laboratory of Membrane Trafficking Mechanisms, Department of Integrative Life Sciences, Graduate School of Life Sciences, Tohoku University, Sendai 980-8578, Japan; ^3^ Department of Biochemistry and Molecular Biology, Graduate School and Faculty of Medicine, The University of Tokyo, Tokyo 113-0033, Japan

**Keywords:** Rab29, Lysosome, Phosphorylation, LRRK2, PKC

## Abstract

Rab proteins are small GTPases that regulate a myriad of intracellular membrane trafficking events. Rab29 is one of the Rab proteins phosphorylated by leucine-rich repeat kinase 2 (LRRK2), a Parkinson's disease-associated kinase. Recent studies suggest that Rab29 regulates LRRK2, whereas the mechanism by which Rab29 is regulated remained unclear. Here, we report a novel phosphorylation in Rab29 that is not mediated by LRRK2 and occurs under lysosomal overload stress. Mass spectrometry analysis identified the phosphorylation site of Rab29 as Ser185, and cellular expression studies of phosphomimetic mutants of Rab29 at Ser185 unveiled the involvement of this phosphorylation in counteracting lysosomal enlargement. PKCα and PKCδ were deemed to be involved in this phosphorylation and control the lysosomal localization of Rab29 in concert with LRRK2. These results implicate PKCs in the lysosomal stress response pathway comprised of Rab29 and LRRK2, and further underscore the importance of this pathway in the mechanisms underlying lysosomal homeostasis.

## Teaser

**Summary:** Rab29 phosphorylation at Ser185 occurs under lysosomal stress and is mediated by PKCα and PKCδ; this phosphorylation regulates lysosome morphology and Rab29 localization to lysosomes together with LRRK2.

## INTRODUCTION

The Rab small GTPases are the largest protein family in the Ras superfamily, with ∼60 proteins identified in mammals (Homma et al., 2021). These Rab GTPases (Rabs) are often called the master regulators of membrane trafficking, modulating the biogenesis, trafficking, tethering or fusion of membranes through various effector proteins. Rab function requires two important modes of regulation. The first is the C-terminal prenylation of Rabs, which happens after synthesis and is responsible for its membrane association. The second is its guanine nucleotide-binding state, which is controlled by guanine nucleotide exchange factors (GEFs) and GTPase-activating proteins (GAPs). GEFs exchange the GDP bound to Rabs to GTP to make Rabs active; GAPs promote hydrolysis of GTP bound to Rabs to make them inactive. Both GEFs and GAPs have a high specificity for each Rab, but activation only occurs on membranes (Bailly et al., 1991; Homma et al., 2021; Sluijs et al., 1992; Waschbüsch and Khan, 2020).

In addition to activation by altering nucleotide-binding states, there are several other means of regulating Rab activity. Among them is phosphorylation, which early studies have shown is associated with altered subcellular localization in (Bailly et al., 1991; Sluijs et al., 1992). This post-translational modification has gained much attention after the discovery of a subset of Rabs being phosphorylated by leucine rich-repeat kinase 2 (LRRK2), a Parkinson's disease (PD)-causative protein (Homma et al., 2021; Steger et al., 2016; Waschbüsch and Khan, 2020). Recently, it has been reported that more than 40 Rab and related proteins are phosphorylated upon activation of the platelet collagen receptor GP6 (Babur et al., 2020), further indicating the importance of Rab regulation by phosphorylation.

Rab29 is one of the Rab proteins identified as being phosphorylated by LRRK2 (Fujimoto et al., 2018; Liu et al., 2018; Steger et al., 2017). *RAB29* is thought to be a risk factor of PD, as it is encoded in the susceptibility locus *PARK16* (Gan-Or et al., 2012; Satake et al., 2009; Tucci et al., 2010), further implicating a link with LRRK2. The consensus is that the main function of Rab29 lies in the regulation of LRRK2 (Kuwahara and Iwatsubo, 2020), with multiple reports observing the activation of LRRK2 (Purlyte et al., 2018) and recruitment of LRRK2 to the Golgi upon overexpression (Liu et al., 2018; Madero-Pérez et al., 2018) or to lysosomes upon lysosomal overload (Eguchi et al., 2018). Other intracellular functions of Rab29 include Golgi-associated trafficking (MacLeod et al., 2013; Wang et al., 2014), AP3-associated protein trafficking (Kuwahara et al., 2016), maintenance of lysosomal homeostasis (Eguchi et al., 2018),and Golgi-independent endolysosomal trafficking (Rivero-Ríos et al., 2020), most of which are functions related to LRRK2. Recent analyses of the direct interaction of these two proteins, with the help of improved structure analysis techniques, have revealed the position and surface of their interaction (McGrath et al., 2021; Vides et al., 2022; Zhu et al., 2022 preprint), further lighting the way to analyze the Rab29-LRRK2 axis.

However, apart from the regulation of LRRK2 by Rab29, the upstream mechanisms regulating Rab29 itself remains largely elusive. We have previously reported that LRRK2-mediated phosphorylation of Rab29 at Ser72 regulates Golgi morphology (Fujimoto et al., 2018). There are also reports that the neighboring Thr71 can also be phosphorylated together (Liu et al., 2018; Steger et al., 2017), which suggests the importance of Rab29 phosphorylation at these sites in steady-state conditions. Under stressed conditions that cause lysosomal overload, which is characterized by increased luminal osmotic pressure and inflation of lysosomes as a result of accumulation of lysosomotropic agents (Eguchi et al., 2018), Rab29 is translocated from Golgi to the lysosomal surface and regulates the morphology of lysosomes (Eguchi et al., 2018). However, the regulatory mechanisms or factors of Rab29 under these conditions are not yet know. Here, we report the discovery of a new phosphorylation of Rab29 at Ser185 that, together with the phosphorylation by LRRK2, regulates the lysosomal stress response, shedding light on further insights into the pathophysiological functions of Rab29.

## RESULTS

### Lysosomal stress leads to LRRK2-independent phosphorylation of Rab29

**Fig. 1.**
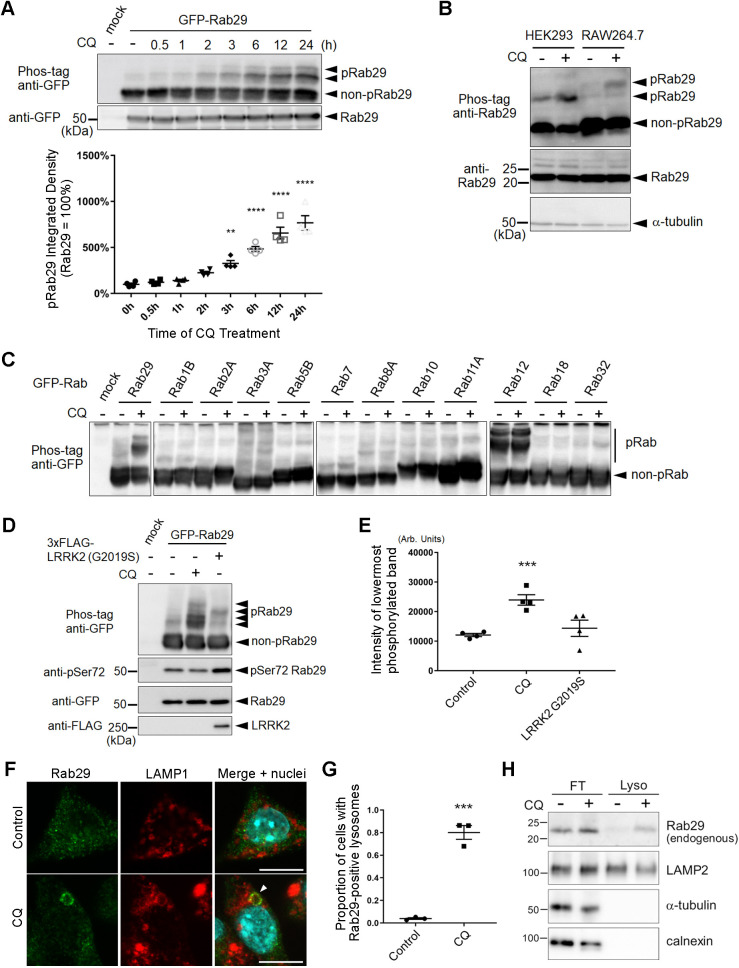
**Lysosomal stress leads to LRRK2-independent phosphorylation of Rab29.** (A) Phosphorylation of Rab29. upon CQ treatment in a time-dependent manner in HEK293 cells overexpressing GFP–Rab29. The images are representative of *n*=4 trials. pRab29 and non-pRab29 indicate phosphorylated and nonphosphorylated Rab29, respectively. Error bars indicate s.e.m.; *n*=4. **P<0.01, ****P<0.0001 [one-way ANOVA followed by Dunnett's test against the control (0 h)]. (B) Phosphorylation of endogenous Rab29 in HEK293 and RAW264.7 cell lines. Representative image of *n*=3 trials. (C) Phosphorylation of a set of Rab proteins with or without CQ in HEK293 cells. A screening of *n*=1 trial. (D) Comparison of Rab29 phosphorylation upon CQ treatment or by LRRK2 in HEK293 cells. Representative image of *n*=4 trials. (E) Quantification of the intensity of the lowermost phosphorylated band in D. Error bars indicate s.e.m.; *n*=4. ***P<0.001 (one-way ANOVA followed by Dunnett's test against the control). (F) Localization of endogenous Rab29 under CQ-treated conditions in RAW264.7 cells. The arrowhead indicates Rab29 colocalization with LAMP1, a lysosomal marker, on enlarged lysosomes. Scale bars: 10 μm. (G) Quantification of lysosomal localization of Rab29 in each field, as shown in F. Error bars indicate s.e.m.; *n*=3 fields. ***P<0.001 (unpaired, two-tailed Student's *t*-test against the control). (H) Biochemical detection of endogenous Rab29 in flow through (FT) and lysosome (Lyso) fractions from HEK293 cells treated with or without CQ. LAMP2, α-tubulin and calnexin were also analyzed as markers of lysosome, cytosol and ER membrane, respectively. Representative image of *n*=3 trials.

We have previously reported that overloading lysosomes causes sequential recruitment of Rab29 and LRRK2 onto enlarged lysosomes (Eguchi et al., 2018) and that this recruitment is brought about by various lysosomotropic agents that cause lysosomal overload (Kuwahara et al., 2020). Therefore, we assumed that there could be a mechanism underlying Rab29 localization to lysosomes under conditions causing lysosomal overload. Chloroquine (CQ), a lysosomotropic agent, was used to elicit lysosomal overload stress in HEK293 cells overexpressing Rab29. We found that Rab29 was phosphorylated upon exposure to CQ, and that the phosphorylation increased over time (Fig. 1A). This phosphorylation was also observed with endogenous Rab29 in HEK293 or RAW264.7 cells (Fig. 1B).

To see whether this phenomenon is prevalent among other members of the Rab family, HEK293 cells expressing various Rab proteins were treated with CQ. Induction of phosphorylation was prominent only in Rab29, but was not observed in any other Rabs tested (Fig. 1C), including Rab32, the closest Rab homolog to Rab29 in the Rab subfamily (Homma et al., 2021), and Rabs 3A, 8A, 10 and 12, which are well-known substrates of LRRK2 (Steger et al., 2016). This difference raised the possibility that LRRK2 is not likely to be the kinase responsible for this Rab29 phosphorylation caused by CQ.

Next, we compared Rab29 phosphorylation mediated by LRRK2 with that by CQ. Co-expression of LRRK2 resulted in phosphorylation as detected in a Phos-tag SDS-PAGE analysis, but its band intensities and patterns were different from those seen upon CQ treatment (Fig. 1D,E). Furthermore, the signal for phospho-Ser72, a residue phosphorylated by LRRK2 (Fujimoto et al., 2018), was unaltered in CQ-treated cells (Fig. 1D), further supporting the LRRK2-independency of the phosphorylation seen upon CQ treatement.

Rab GTPases require prenylation at its C-terminus to localize to the membrane and deliver their function (Homma et al., 2021). This can be blocked by statins via deprivation of geranylgeranyl diphosphate, the precursor of the C-terminal prenylation, from cells (Binnington et al., 2016; Gomez et al., 2019). Treatment with lovastatin in addition to CQ resulted in an inhibition of the phosphorylation in Rab29 (Fig. S1A,B), suggesting the requirement of membrane localization for this phosphorylation.

Also in our previous study, we found that Rab29 overexpression facilitated the translocation of LRRK2 and overexpressed Rab29 itself to enlarged lysosomes under CQ exposure, and the knockdown of Rab29 suppressed the translocation of LRRK2, indicative of its function upstream of LRRK2 (Eguchi et al., 2018). We therefore assumed that the localization of endogenous Rab29 might also change in the same manner as LRRK2. Immunocytochemical analysis revealed that CQ treatment caused the translocation of endogenous Rab29 to enlarged lysosomes in RAW264.7 cells (Fig. 1F). A similar translocation was also detected in other cell types, including HeLa cells, HEK293 cells, human lung epithelial A549 cells and mouse microglial MG6 cells (Fig. S2A–D). This translocation of Rab29 was observed frequently, but not in all cells, and the proportion of cells with Rab29-positive lysosomes was comparable to that reported previously for LRRK2 (Eguchi et al., 2018) (Fig. 1G). Furthermore, enrichment of endogenous Rab29 in lysosomal fraction upon CQ treatment was confirmed by biochemical isolation of lysosomes using dextran magnetite (Fig. 1H).

### Phosphorylation of Rab29 could occur on the lysosomal surface

**Fig. 2.**
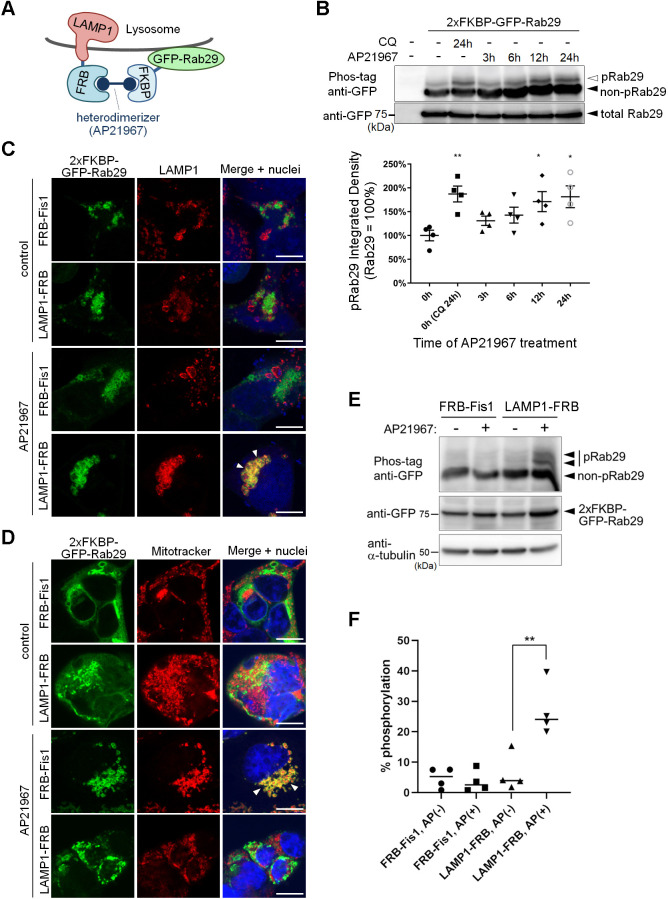
**Phosphorylation of Rab29 could occur on the lysosomal surface.** (A) A scheme of the forced localization technique used in this study. Upon treatment with the heterodimerizer AP21967, FKBP-bound Rab29 is anchored away to FRB-positive compartments in the cell. (B) Phosphorylation of FKBP-Rab29 over time upon forced lysosomal localization by AP21967 in HEK293 cells co-expressing LAMP1–FRB. The images are representative of *n*=4 trials. Error bars indicate s.e.m.; *n*=4. *P<0.05, **P<0.01 [one-way ANOVA followed by Dunnett's test against the control (0 h)]. (C,D) Anchoring Rab29 at mitochondria (Fis1) or lysosomes (LAMP1) in HEK293 cells stained with (C) a LAMP1 antibody or (D) MitoTracker Red. Arrowheads indicate Rab29 colocalized with (C) LAMP1 or (D) mitochondria. Scale bars: 10 μm. (E) Phosphorylation of Rab29 upon forced localization to lysosomes (LAMP1) but not to mitochondria (Fis1) in HEK293 cells. Representative image of *n*=4 trials. (F) Quantification of the phosphorylated bands in E. The percentage of Rab29 phosphorylation (pRab29) was calculated by dividing the intensities of bands indicating pRab29 by the sum of those indicating non-pRab29 and pRab29. *n*=4. **P<0.01 (one-way ANOVA followed by Tukey's test).

Given that CQ exposure caused Rab29 phosphorylation and its translocation to enlarged lysosomes, we speculated that this phosphorylation could occur at the lysosomal membrane. To explore this possibility, we utilized a forced intracellular protein translocation system (Belshaw et al., 1996), a drug-inducible method based on the heterodimerization of the FK506-binding protein (FKBP) and the FKBP-rapamycin binding domain (FRB) in the presence of rapamycin analogs (Fig. 2A). FKBP-tagged Rab29 expressed in HEK293 cells was forced to localize at the lysosomal surface by co-expressing the lysosomal protein LAMP1–FRB followed by treatment with AP21967, a rapamycin analog heterodimerizer. Similar to what was seen with CQ treatment, phosphorylation of Rab29 increased over time upon treatment with AP21967 (Fig. 2B).

We confirmed that the intracellular localization of Rab29 changed to lysosomes when LAMP1–FRB was co-expressed and cells treated with AP21967 (Fig. 2C). By contrast, when the mitochondrial protein FRB–Fis1 was co-expressed instead of LAMP1–FRB, Rab29 was not localized to lysosomes but recruited to the mitochondria (Fig. 2D). We found that the co-expression of LAMP1–FRB, but not FRB–Fis1, induced the phosphorylation of Rab29 (Fig. 2E,F), indicating that the phosphorylation of Rab29 could occur on the lysosomal surface.

### The Ser185 residue of Rab29 is phosphorylated under lysosomal stress

**Fig. 3.**
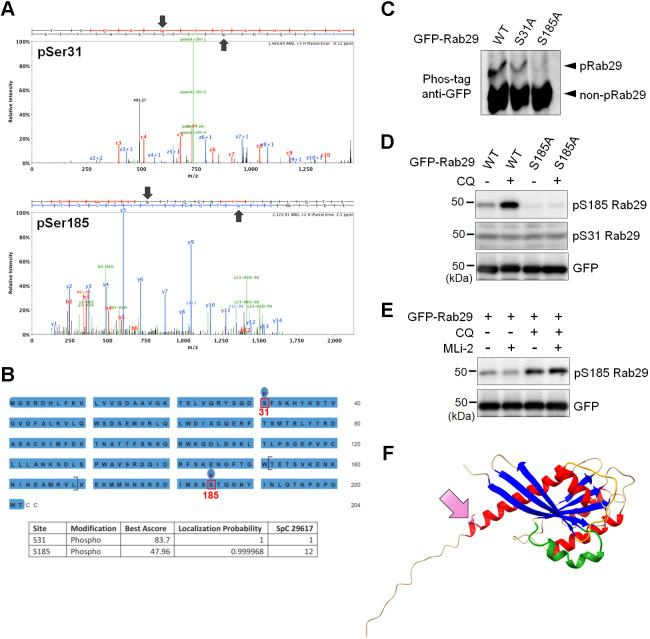
**Ser185 residue of Rab29 is phosphorylated under lysosomal stress.** (A) A representative spectrum of LC-MS/MS indicating phosphorylation at Ser31 or Ser185. (B) A sequence of Rab29 showing the putative phosphorylation sites Ser31 and Ser185. (C) Reduced phosphorylation of Rab29 upon alanine substitution of Ser185, but not Ser31, under CQ treatment in HEK293 cells. Representative image of *n*=5 trials. (D) Confirmation of Ser185 phosphorylation by using phospho-specific antibodies against HEK293 cell samples. Representative image of *n*=3 trials. (E) No changes in CQ-induced phosphorylation of Rab29 by inhibition of LRRK2 in HEK293 cells. Representative image of *n*=3 trials. (F) An AlphaFold2-generated structural image of Rab29. The pink residue pointed to by the pink arrow is Ser185. The orange and green chains indicate switch 1 and 2, respectively. α-Helices are colored in red and β-sheets in blue.

Given that we assumed that the phosphorylation we found occurs independently of LRRK2, we sought to determine the exact phosphorylation site of Rab29. After pulling down GFP–Rab29 from CQ-treated cells, phosphorylated Rab29 was separated by Phos-tag SDS-PAGE and analyzed by mass spectrometry, which identified the putative phosphorylation sites as Ser31 or Ser185 (Fig. 3A,B). Ser31 is just before the switch 1 region, and Ser185 is in the third complementarity-determining region (CDR3) (Stein et al., 2012; Waschbüsch and Khan, 2020), which is a motif that contributes to the specificity of its effector (Ostermeier and Brunger, 1999). Both sites harbor a potential to regulate the activity of this GTPase.

To further determine the phosphorylation sites, serine to alanine mutants [Ser31 to alanine (S31A) or Ser185 to alanine (S185A)] were overexpressed in HEK293 cells followed by treatment with CQ. The Phos-tag SDS-PAGE revealed a major decrease of the phosphorylated Rab29 band in the S185A mutant, compared to either S31A or wild-type (WT) Rab29 (Fig. 3C). Thus, the phosphorylation of Rab29 under CQ treatment is likely to be at Ser185.

To further examine the Rab29 phosphorylation, we developed a phospho-Ser185 specific rabbit polyclonal antibody and analyzed Rab29-overexpressing cells. We found that the signal recognized by this antibody was elevated upon CQ exposure and was abolished in the S185A mutant (Fig. 3D). Treatment with a LRRK2-specific kinase inhibitor, MLi-2, did not affect the band intensity of phospho-Ser185 (Fig. 3E), confirming that this phosphorylation is indeed independent of LRRK2.

According to a structural prediction by AlphaFold2 (Jumper et al., 2021), the Ser185 residue is located at the end of the last helix (Fig. 3F). As this site lies in CDR3 (Stein et al., 2012; Waschbüsch and Khan, 2020) and phosphorylations in this region could alter binding with GEFs or guanine nucleotide dissociation inhibitors (GDIs) (Waschbüsch and Khan, 2020), it seemed reasonable to assume that this phosphorylation could somehow regulate the function of Rab29.

### Phosphomimetics of Rab29 Ser185 alleviated CQ-induced lysosomal enlargement

**Fig. 4.**
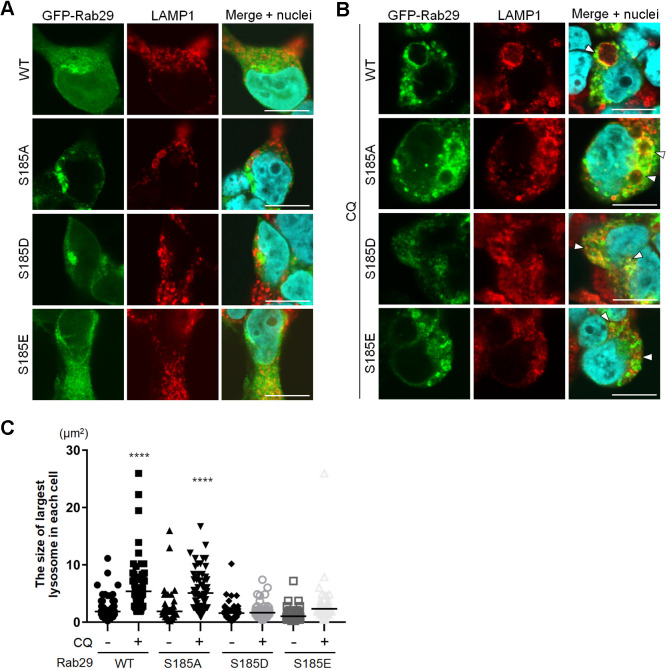
**Phosphomimetics of Ser185 alleviate CQ-induced lysosomal enlargement.** (A) Lysosome morphology and Rab29 localization in HEK293 cells expressing GFP–Rab29 WT or Ser185 mutants at steady state. (B) Lysosome morphology and Rab29 localization in HEK293 cells expressing GFP–Rab29 WT or mutants upon 8 h of CQ treatment. Arrowheads indicate lysosomes with Rab29 accumulation. Scale bars: 10 μm. (C) Statistical analysis of lysosomal size in A and B. Each shape shows the area of the largest lysosome in each cell, obtained by elliptical approximation of each immunocytochemistry image. Only lysosomes from all of the Rab29-positive cells were included in this analysis (92–172 cells in each condition). The mean is shown by a black horizontal bar in each sample. ****P<0.0001 [one-way ANOVA followed by Dunnett's test against the control (wild type, no CQ) sample].

We next sought to analyze the role of this phosphorylation in terms of lysosomal regulation. Phosphorylation of the Rab29 Ser185 residue was mimicked by replacement with aspartate or glutamate (S185D or S185E, respectively), or disabled by alanine substitution (S185A). WT or these mutants tagged with GFP were overexpressed in HEK293 cells, and their effects on Rab29 localization or lysosome morphology were assessed. Under steady-state conditions (i.e. without CQ), overexpression of WT or these mutants did not alter their subcellular localization, and the morphology of lysosomes remained small and unaltered (Fig. 4A). Upon CQ treatment, WT or S185A Rab29-overexpressing cells exhibited enlarged lysosomes, as observed in previous studies (Eguchi et al., 2018), whereas S185D or S185E Rab29-overexpressing cells exhibited smaller lysosomes, similar to those observed in CQ-untreated samples (Fig. 4B,C). This was also confirmed by correlative light and electron microscopy (CLEM), as LAMP1-positive lysosomes bearing S185D or S185E mutant Rab29 were indeed smaller under electron microscopy (Fig. S3). As we had shown previously that depletion of Rab29 produces heavily enlarged lysosomes upon CQ treatment (Eguchi et al., 2018), the smaller lysosomes seen under the S185D or S185E overexpression were considered to be formed through a dominant active effect of these proteins.

LRRK2 inhibition or knockdown has been shown to increase lysosomal size when cells are treated with CQ (Eguchi et al., 2018; Kuwahara et al., 2020). Also, several studies have reported that overexpression of Rab29 increases the LRRK2 kinase activity (Kuwahara et al., 2020; Purlyte et al., 2018). Therefore, we next examined whether any of these mutant Rab29 forms altered the LRRK2 kinase activity. Using Rab10 phosphorylation at Thr73 as a readout (Eguchi et al., 2018; Ito et al., 2016; Kuwahara et al., 2020), LRRK2 kinase activity was measured and no significant changes were observed (Fig. S4A,B). Also, no changes in LRRK2 binding to Rab29 mutants compared to WT Rab29 were observed (Fig. S4C).

Also, an AlphaFold2 prediction of Rab29 phosphomimetics showed an ‘open’ conformation around Ser72 (Fig. S5A), so we decided to assess whether these phosphomimetic Rab29 were phosphorylated more at Ser72 due to the possibly increased accessibility of this residue. However, no change in phosphorylation of Ser72 was observed either with (Fig. S5B) or without CQ treatment (Fig. S5C). These data together suggest that phosphorylation at Ser185 does not affect LRRK2 in any way observed.

### PKCs are involved in Ser185 phosphorylation and lysosomal localization of Rab29

We next addressed the question as to which kinase is responsible for this phosphorylation. Kinase determination could be accomplished by computer-based prediction and followed by confirmation in *in vitro* experiments. An attempt to predict a kinase for the Ser185 of Rab29 using NetPhos3.1 (Blom et al., 1999) was made, but no candidates that were scored strongly enough to likely to be a candidate (Table S1).

**Fig. 5.**
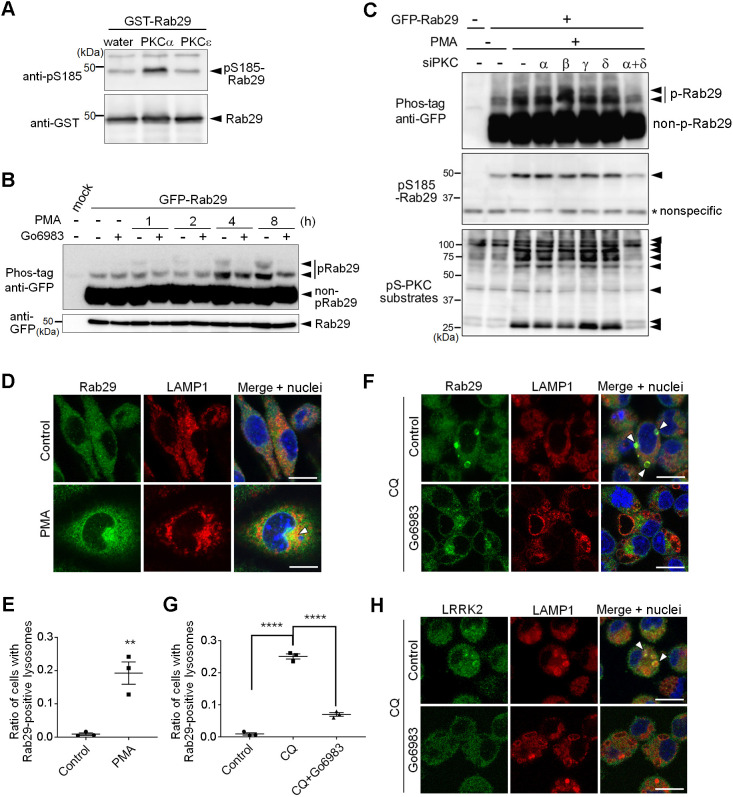
**PKCs are involved in Ser185 phosphorylation and lysosomal localization of Rab29.** (A) *In vitro* kinase assay using recombinant Rab29 and PKCα or PKCε. (B) Phosphorylation of Rab29 with PMA or Go6983 over time in HEK293 cells. (C) Cells were treated with siRNAs for PKC isozymes that were targeted by both PMA and Go6983, and the phosphorylation of Rab29 upon PMA treatment (24 h) was assessed by Phos-tag or anti-phospho-S185 antibody. Overall PKC activity was additionally monitored by detecting phospho-Ser PKC substrates (pS-PKC substrates). Images in A–C are representative of *n*=3 trials. (D) Rab29 localization upon PMA treatment in RAW264.7 cells. The arrow indicates colocalization of endogenous Rab29 with LAMP1. Scale bars: 10 μm. (E) Quantification of lysosomal localization of Rab29, as shown in D. Error bars indicate s.e.m.; *n*=3 fields **P<0.01 (unpaired two-tailed *t*-test). (F) Lysosomal localization of endogenous Rab29 upon CQ and Go6983 treatment in RAW264.7 cells. Arrowheads indicate enlarged lysosomes with Rab29 accumulation. Scale bars: 10 μm. (G) Quantification of lysosomal localization of Rab29, as shown in F. Error bars indicate s.e.m.; *n*=3 fields. ****P<0.0001 (one-way ANOVA followed by Tukey's test). (H) Lysosomal localization of endogenous LRRK2 upon CQ and Go6983 treatment in RAW264.7 cells. Arrowheads indicate enlarged lysosomes with Rab29 accumulation. Images in H are representative of three experiments. Scale bars: 10 μm.

To date, there have been some reports on Rab serine/threonine phosphorylation concerning its C-terminal region. Rab1 and Rab4 are reported to be phosphorylated by CDK1 (Bailly et al., 1991), Rab9 by Ulk1 (Saito et al., 2018), and Rab11 and Rab37 by PKCα (Pavarotti et al., 2012; Tzeng et al., 2017; reviewed in Waschbüsch and Khan, 2020 and Homma et al., 2021). Since PKCα was the only hit in the NetPhos3.1 and was listed as a kinase that phosphorylates Rabs at the C-terminus, we reasoned that this might be a kinase responsible for the phosphorylation of Rab29. Using PKCε as a negative control, we incubated recombinant Rab29 either with PKCα or PKCε *in vitro*. Only Rab29 mixed with PKCα exhibited a band indicative of phosphorylation at Ser185 (Fig. 5A), suggesting PKCα acts as a kinase for Rab29.

Phorbol 12-myristate 13-acetate (PMA) is a renowned activator of several PKC isoforms including PKCα. Treatment of HEK293 cells overexpressing Rab29 with PMA resulted in the increase of the levels of phosphorylated Rab29 over time (Fig. 5B). This phosphorylation was prevented upon treatment with a PKC inhibitor Go6983 (Fig. 5B). Because Go6983 targets several PKC isoforms (i.e. PKCα, PKCβ, PKCγ and PKCδ; Gschwendt et al., 1996), we knocked down these four subtypes of PKC and examined PMA-induced phosphorylation of Rab29. We could detect the increase of Ser185 phosphorylation, and this was inhibited by a combined knockdown of PKCα and PKCδ, although none of them showed any effect when knocked down individually (Fig. 5C). As phosphorylations of other PKC substrates were also largely suppressed by a combined knockdown of PKCα and PKCδ (Fig. 5C), these two PKC isoforms are considered to act dominantly and compensate for each other during phosphorylation of Rab29 and other substrates in these cells.

As changes in Rab29 localization to the lysosomal surface resulted in its phosphorylation (Fig. 2E), we next assessed whether phosphorylation of Rab29 in cells could change in its localization. Treatment of RAW264.7 cells with PMA resulted in the translocation of endogenous Rab29 to lysosomes (Fig. 5D,E). In contrast, inhibition of PKC by Go6983 resulted in a diminution in CQ-induced translocation of endogenous Rab29 to lysosomes (Fig. 5F,G). This effect of Go6983 on Rab29 translocation was also confirmed by biochemical isolation of lysosomes (Fig. S6). These data suggest that the PKC-mediated phosphorylation of Rab29 localizes Rab29 to lysosomes. Also, considering earlier data that the phosphorylation occurs upon forcing the localization of Rab29 to lysosomes, it seems likely that Rab29 is stably trapped on lysosomal membranes once phosphorylated. Furthermore, translocation of LRRK2 upon CQ treatment and its inhibition by Go6983 were observed to occur in a similar manner to in the case of Rab29 (Fig. 5H), supporting the link between PKC and LRRK2 via Rab29.

### LRRK2 is also a regulator of Rab29 localization

**Fig. 6.**
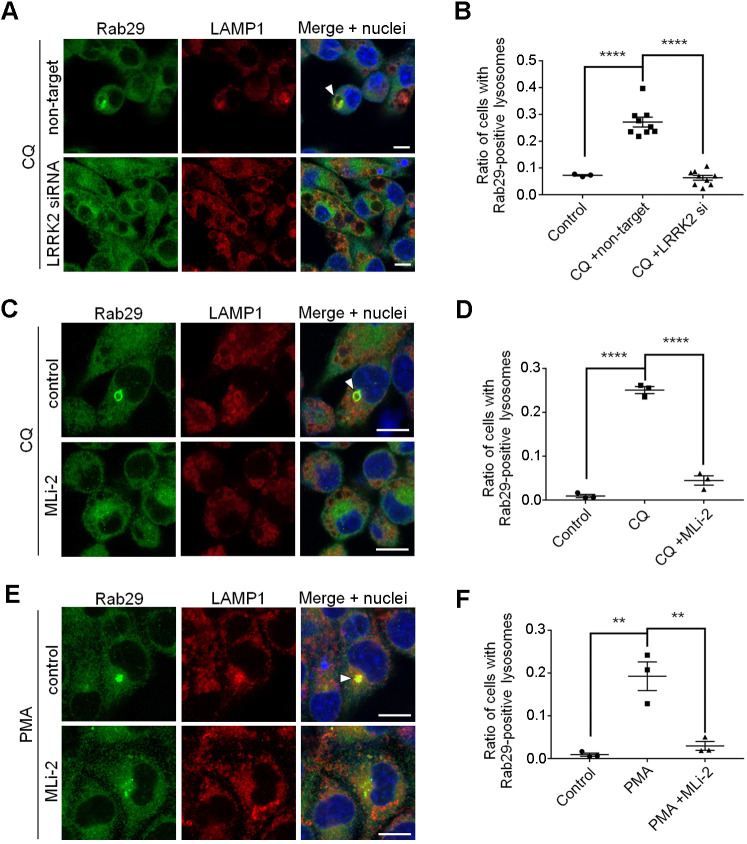
**LRRK2 is also a regulator of Rab29 localization.** (A) Rab29 localization upon knockdown of LRRK2 and CQ treatment in RAW264.7 cells. The arrowhead indicates enlarged lysosomes with Rab29 accumulation. (B) Quantification of lysosomal localization of Rab29, as shown in A. Error bars indicate s.e.m.; *n*=3, 9, 9 fields, respectively. ****P<0.0001 (one-way ANOVA followed by Tukey's test). (C) Rab29 localization upon MLi-2 (a LRRK2 inhibitor) and CQ treatment in RAW264.7 cells. The arrowhead indicates enlarged lysosomes with Rab29 accumulation. (D) Quantification of lysosomal localization of Rab29, as shown in C. Error bars indicate s.e.m.; *n*=3 fields. ****P<0.0001 (one-way ANOVA followed by Tukey's test). (E) Rab29 localization upon MLi-2 and PMA treatment in RAW264.7 cells. The arrowhead indicates enlarged lysosomes with Rab29 accumulation. (F) Quantification of lysosomal localization of Rab29, as shown in E. Error bars indicate s.e.m.; *n*=3 fields. **P<0.01 (one-way ANOVA followed by Tukey's test). Scale bars: 10 μm.

To confirm that Rab29 functions upstream of LRRK2, we knocked down the expression of LRRK2 and analyzed the localization of endogenous Rab29 upon CQ treatment. Contrary to expectations, however, knockdown of LRRK2 caused a lessened accumulation of Rab29 to enlarged lysosomes (Fig. 6A,B; Fig. S7). LRRK2 kinase inhibition by MLi-2 also resulted in a lower level of localization of Rab29 to CQ-induced enlarged lysosomes (Fig. 6C,D), and this was further confirmed by biochemical isolation of lysosomes (Fig. S6). These results and our previous finding that Rab29 regulates LRRK2 localization (Eguchi et al., 2018) together indicate that, during CQ exposure, LRRK2 is regulated by Rab29, but also acts as a regulator of Rab29 localization in the opposite direction.

To see whether LRRK2 controls Rab29 localization in other conditions, we turned to PMA treatment, which we found caused Rab29 to localize to perinuclear lysosomes without inducing lysosomal enlargement. Translocation of endogenous Rab29 upon PMA treatment was also suppressed by LRRK2 kinase inhibition, as seen in CQ-treated cells (Fig. 6E,F). These results further support the idea that the translocation of Rab29 requires both phosphorylations mediated by LRRK2 and PKCs.

## DISCUSSION

**Fig. 7.**
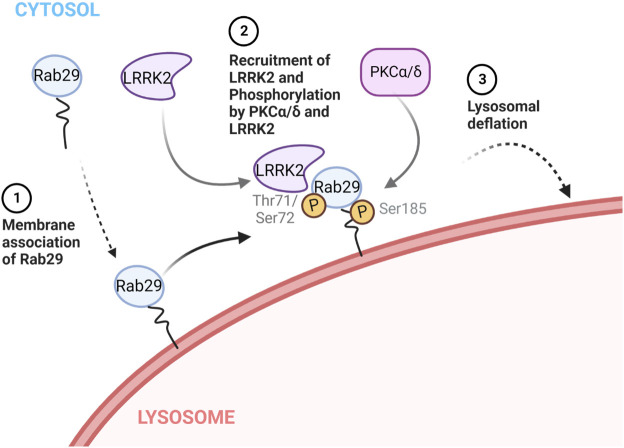
**A model for Rab29 translocation, phosphorylation and their effects under lysosomal stress.** Upon stimuli that cause lysosomal overload, (1) Rab29 first associates with the lysosomal membranes, and (2) Rab29 recruits LRRK2 to lysosomal membranes and is phosphorylated by PKCα and/or PCKδ, and LRRK2, which stabilizes Rab29 on lysosomal membranes. Then, (3) Rab29 and LRRK2 on lysosomes induces downstream effects that lead to lysosomal deflation. This figure was created with BioRender.com.

Phosphorylation of Rab proteins is a common but noteworthy post-translational modification that allows quick regulation and alteration of their functions. However, phosphorylation at the C-terminal region of Rabs is not very common, with only six instances of such described in literature where the kinase responsible has been identified (Homma et al., 2021; Waschbüsch and Khan, 2020). Rab11 and Rab37 are reported to be phosphorylated by PKCα, Rab7a by Src, Rab9 by Ulk1, and Rab1 and Rab4 by Cdc2 (Bailly et al., 1991; Lin et al., 2017; Pavarotti et al., 2012; Saito et al., 2018; Tzeng et al., 2017). Here, we provided evidence that Rab29 is phosphorylated by PKCα and PKCδ, which could occur on the lysosomal membrane and counteract lysosomal overload elicited by the lysosomotropic compound CQ. We also showed that Rab29 localization to lysosomes depends on the phosphorylations mediated by the PKCs as well as LRRK2, a known Rab29 interactor. Notably, Rab29 phosphorylation also occurred after Rab29 was forced to localize lysosomes (Fig. 2B,E), and considering that both PKCs and LRRK2 are known to be active on membranes (Gomez et al., 2019; Prevostel et al., 2000), and that phosphorylation of Rab29 is more likely to happen on the membranes than in the cytosol (Liu et al., 2018), it is likely that the phosphorylation of Rab29 by these kinases result in Rab29 becoming ‘trapped’ on the lysosomal membrane. Although the plausible effectors of Rab29 are yet to be discovered, this novel phosphorylation seems to have a function in counteracting lysosomal stress in coordination with LRRK2 and PKCs (Fig. 7).

There have been several reports concerning the relationship between Rab29 and LRRK2, mainly about the phosphorylation and recruitment. The consensus from past literature is that LRRK2 phosphorylates Rab29 (at Thr71 and Ser72; Fujimoto et al., 2018; Liu et al., 2018; Steger et al., 2017), and that Rab29 recruits and activates LRRK2 (either at the Golgi or lysosomes; Eguchi et al., 2018; Purlyte et al., 2018). The exact mechanism of LRRK2 activation also is unclear, but it is possibly mediated by heteromultimer formation with Rab29 and/or by membrane association of LRRK2 (Kluss et al., 2022; Zhu et al., 2022 preprint). Although a closer investigation into the order of events that we discovered is needed, our data suggest that the regulation of the localization of Rab29 and LRRK2 is intertwined, both needing each other to be stably localized on membranes. A recent report showed that LRRK2 has two different sites to which Rabs can bind, one for unphosphorylated Rabs, and one exclusively for phosphorylated Rab8 or Rab10 (Vides et al., 2022). Inhibition of LRRK2 kinase activity caused a Rab29 localization to become dispersed (Fig. 6C,D), so it could be reasoned that the binding of LRRK2-phosphorylated Rab29 to LRRK2 at multiple sites is necessary for the localization or stabilization of Rab29 on the lysosomal membrane.

PKCα and PKCδ are ubiquitously expressed kinases implicated in a multitude of pathways from cell proliferation to apoptosis (Black et al., 2022; Singh et al., 2017). They are mainly localized at the plasma membrane, where they get activated by diacylglycerol. Both PKCs are reported to be transported to the endosomal pathway, while keeping their active state, upon PMA treatment (Bailey et al., 2014; Prevostel et al., 2000). Hence, the perinuclear localization of Rab29 that overlapped with LAMP1 upon PMA treatment (Fig. 5D) could be explained in this context – that active PKCα or PKCδ happened to encounter Rab29 somewhere on the endosomal pathway and the resulting phosphorylation caused the stabilization of its localization there. Of course, we do not rule out the possibility that there could be an endomembrane regulation by these PKCs that results in a localization change of Rab29. We should also note that the major PKC isoform involved in Rab29 phosphorylation might vary depending on cell type; a combined knockdown of PKCα and PKCδ in our HEK293 cells suppressed the phosphorylation of many substrates, so it is possible that the phenomenon might not be specific to Rab29 and that other PKCs might work in other cell types where that isoforms are more dominant. Another note is that our attempts to detect *in vitro* phosphorylation of Rab29 by PKCδ were not successful, unlike for PKCα. The reason for the difference compared to the results observed in cells is unknown, but *in vitro* environment is more artificial, and even if *in vitro* results were as expected, it is still unclear whether PKCs really directly phosphorylate Ser185 in cells. Thus, the possibility remains that PKCs phosphorylate Rab29 both directly and indirectly in cells.

The exact mechanism of Rab29 localization to LAMP1-positive compartments, especially how Rab29 gets ‘trapped’ on the membrane where it is or has been phosphorylated, is still unclear. Usually, Rab localization is strictly regulated by their GEFs, and further investigation is needed to answer whether this phenomenon is also due to a GEF and its activity. Recently, Rabaptin5 was proposed to be a GEF for Rab29 (Shrivastava et al., 2022), and it might be worthwhile to assess their interactions in similar conditions. There might also exist unknown interactors specific to Rab29 phosphorylated at Ser185 that mediate Rab29 localization.

We have previously reported that the knockdown of Rab29 causes enhanced enlargement of lysosomes and impaired release of lysosomal contents upon exposure to CQ (Eguchi et al., 2018). The amelioration of lysosomal enlargement with phosphomimetic Rab29 overexpression (Fig. 4B,C) could be explained as the reverse of knockdown, and lysosomal content release downstream of LRRK2 could have had an effect on regulating the size of lysosomes. We were not able to assess this possibility due to technical difficulties concerning cell types.

Recently, inhibition of PKC has been reported to prevent aggregation of α-synuclein, a key causative protein of PD, upon transfection with pre-aggregated ‘seed’ α-synuclein (Svanbergsson et al., 2021). In our data, PKC inhibition resulted in dispersed Rab29 localization under lysosomal stress (Fig. 5F). Considering that most pathogenic LRRK2 mutations lead to increased phosphorylation of its substrates, either by upregulation of kinase activity or enhanced substrate binding, and that Rab29 activates LRRK2, our data is in line with this view and might further link the abnormalities in Rab29 with PD in terms of pathological mechanisms. This also highlights the importance of analyzing the detailed functions of Rab29 at lysosomes where it could respond to intracellular stress.

In summary, our data provide evidence for a novel phosphorylation in Rab29 that involves PKCs and is capable of controlling Rab29 localization in concert with LRRK2. These phosphorylations and the following localization change were considered to be important for maintaining lysosomal morphology upon lysosomal overload stress. Further studies would be needed to clarify how this stress response mechanism is initiated, whether it is regulated by other key molecules and whether it could be involved in the pathogenesis of PD.

## MATERIALS AND METHODS

### Antibodies

The antibodies against the following proteins were used in this study: GFP [Thermo Fisher Scientific, A11122, 1:1000 for western blotting (WB); MBL, 598, 1:500 for immunocytochemistry (ICC)], LAMP1 (BD Pharmingen, H4A3, 1:500 for ICC, 1:1000 for WB), phospho-Ser72-Rab29 [generated in our previous study (Fujimoto et al., 2018), 1:100 for WB], FLAG (MBL, FLA-1, 1:10,000 for WB), LRRK2 (MJFF, MJFF2, 1:250 for ICC, 1:1000 for WB), α-tubulin (Abcam, DM1A, 1:10,000 for WB), non-phospho-Ser185-Rab29 (generated in this study, 1:1000 for WB), phospho-Ser185-Rab29 (generated in this study, 1:1000 for WB), LAMP1 (Bio-Rad, 1D4B, 1:1000 for ICC and WB), Rab10 phospho-Thr73 (Abcam, MJF-R21, 1:1000 for WB), Rab10 (Cell Signaling Technology, D36C4, 1:1000 for WB), Rab29 (Abcam, MJF-R30-124, 1:250 for ICC, 1:1000 for WB) and GST (GE Healthcare, 27-4577-01, 1:2000 for WB). Secondary antibodies for immunocytochemistry were goat or donkey anti-IgGs labeled with Alexa Fluor 488, Alexa Fluor 546, Alexa Fluor 555 or Alexa Fluor 647 (Thermo Fisher Scientific, 1:500). Secondary antibodies for immunoblotting were HRP-labeled anti-IgGs (Jackson Immunoresearch, 1:10,000).

### Reagents

The following reagents were used at final concentrations as indicated: xhloroquine (CQ) (50 μM, Sigma-Aldrich), phorbol 12-myristate 13-acetate (PMA) (100 nM, Santa Cruz Bioctechnology), MLi-2 (50 nM, Abcam), Go6983 (200 nM, Sigma-Aldrich), AP21967 (1 μM, TaKaRa) and MitoTracker Red CMXRos (100 nM, Cell Signaling Technology).

### Generation of phospho-Ser185-Rab29 specific antibodies

To generate human phospho-Ser185-Rab29 specific antibody, rabbits were immunized with KLH-conjugated peptides (KLH-RNSTEDIMSL(pS)TQGD, KLH-RNSTEDIMSLSTQGD, human Rab29 sequences around Ser185) three times with 2-week intervals. Serum was collected 6 weeks after the final immunization, and purified by dual affinity purification, which is a method using non-phosphorylated peptides as a first column and purifying the flow-through with phosphorylated peptides, ensuring more affinity to phosphorylated peptides. These processes were performed at GL Biochem Ltd. (Shanghai, China)

### Plasmids and siRNA

The plasmid pEGFP-C1-human Rab29 was generated by inserting the human Rab29 sequence from pFN21A-Halo-Rab29 (Promega, #FHC08084) into the BglII–EcoRI site of pEGFP-C1-rat Rab29 plasmid that was used previously (Fujimoto et al., 2018). Phospho-mutants of Rab29 were generated by a site-directed PCR mutagenesis protocol. A set of plasmids encoding EGFP-mouse Rabs was prepared as described previously (Tsuboi and Fukuda, 2006). LAMP1–FRB and FRB–Fis1 plasmids were provided by Dr. Richard J. Youle (NIH, Bethesda, MD, USA) (Lazarou et al., 2012). The plasmid encoding 2×FKBP-GFP-Rab29 was generated by transferring 2×FKBP sequence from Addgene plasmid #20149 into HindIII site of pCMV10 plasmid (provided by Dr Genta Ito, Teikyo University, Japan) followed by inserting the EGFP–Rab29 sequence into NotI-XhoI site of pCMV10. The siRNAs used were purchased from Dharmacon (siGenome smart pool) The target and catalog IDs are as follows: Lrrk2 (mouse): M-049666-01-0005; PRKCA (PKCα, human): M-003523-03-0005; PRKCB (PKCβ, human): M-003758-04-0005; PRKCG (PKCγ, human): M-004654-01-0005; and PRKCD (PKCδ, human): M-003524-01-0005.

### Cell culture and transfection

The human embryonic kidney cell line HEK293 (purchased from ATCC, cat. no. CRL-1573), mouse macrophage-like cell line RAW264.7 (purchased from ECACC, cat. no. 91062702), and human tumor-derived cell line HeLaM cells (referred to as HeLa cells throughout this study; RIKEN BRC, cat. no. RCB5388) and human adenocarcinoma derived cell line A549 (purchased from ECACC, cat. no. 86012804) were cultured in Dulbecco's modified Eagle's medium (DMEM; Sigma-Aldrich) with 10% Fetal bovine serum (FBS; Biowest) and 1% penicillin-streptomycin (PS, Gibco) under 5% CO_2_ at 37°C. The mouse microglial cell line MG6 (from RIKEN BRC, cat. no. RCB2403; Nakamichi et al., 2006; Takenouchi et al., 2005) was cultured in the conditions and medium described above with an addition of 10 μg/ml human insulin and 0.1 mM 2-mercaptoethanol. RAW264.7 and MG6 cells were maintained on a Petri dish for suspension cell culture (Sumitomo Bakelite), passaged by pipetting off attached cells and were activated by treatment with IFN-γ (15 ng/ml, Cell Signaling) at 24–48 h prior to analysis. All other cells were maintained on a tissue culture-treated culture dish (Corning) and passaged by trypsin digestion and pipetting. Each of the cell lines was tested negative for contamination of mycoplasma.

Transfection of plasmid vectors or siRNAs to HEK293 cells was conducted using Lipofectamine 2000 (Thermo Fisher Scientific) according to the manufacturer's recommended protocols. Transfection of siRNA to RAW264.7 cells was conducted using Lipofectamine RNAiMAX (Thermo Fisher Scientific) according to the manufacturer's recommended protocols for reverse transfection.

### Treatment of cells with reagents

HEK293, HeLa and A549 cells were treated with each reagent for 24 h unless otherwise stated. RAW264.7 and MG6 cells were treated with each reagent for 3 h. The final concentrations are as described in the events section unless otherwise stated.

### Cell culture and fixation for immunocytochemistry

Cells intended for ICC were cultured on cover glasses (Matsunami) coated with poly-D-lysine (PDL). Coating was performed by incubating 200 μl of PDL, diluted to 50 μg/ml with Dulbecco's phosphate-buffered saline (DPBS), on each cover glass for more than 30 min at 37°C, then washed several times with DPBS. Cover glasses were washed two times with 1 M NaOH for an hour each, then washed with 70% ethanol for two times or more and stored in 70% ethanol at 4°C.

Fixing of cells was conducted by immersing cover glasses in 4% paraformaldehyde (PFA) in DPBS for 30 min at room temperature. After fixation, cover glasses were washed with DPBS and immersed in 100% ethanol for sample dehydration at −20°C. Samples were stored in this state until used for immunocytochemistry.

For cells with mitochondrial staining with MitoTracker Red, cells were fixed with 100% methanol at −20°C for 15 min. After fixation, cover glasses were washed with DPBS.

### Immunocytochemistry

Samples stored in 100% ethanol were washed with DPBS before blocking with blocking buffer [3% (w/v) BSA, 0.1–0.5% Triton X-100 in DPBS] for 30 min at room temperature. Samples were then incubated with primary antibodies diluted in blocking buffer for 3 h at room temperature or overnight at 4°C. Each cover glass was placed on a 35 μl spot of diluted primary antibody solution made on a sheet of parafilm (Bemis) so that the surface with cells present is facing the antibody solution (Sakurai and Kuwahara, 2021). After washing with DPBS three times for 5 min, samples were incubated with secondary antibodies [Alexa-conjugated anti-IgG (Thermo Fisher Scientific), 1:500 dilution] mixed with the nuclear staining agent DRAQ5 (Biostatus, 1:2000) or DAPI (Thermo Fisher Scientific, 1:2000) for 1 h at room temperature or overnight at 4°C. After three further washes by DPBS, samples were mounted on slide glasses (Matsunami) with 7 μl of Permafluor mountant (Thermo Fisher) or ProLong Diamond antifade mountant (Thermo Fisher Scientific) for each cover glass.

### CLEM

HEK293 cells intended for CLEM were cultured on glass base dishes with 150-μm grids (TCI-3922-035R-1CS, Iwaki) coated with PDL as above. Cells were fixed by 2% PFA (EM grade, Nacalai Tesque) and 0.5% glutaraldehyde (TAAB) in 0.09 M phosphate buffer (pH 7.4) for 1 h at room temperature. The cells were washed three times with 0.09 M phosphate buffer and fluorescence microscopy images were acquired via a confocal microscope (FV1000, Olympus). Sample preparation and acquisition of electron microscopy images were undertaken as in a previous report (Takahashi et al., 2022).

### Preparation of cell lysates

Cultured cells were washed with DPBS, then scraped off in lysis buffer [50 mM Tris-HCl pH 7.6, 150 mM NaCl, 0.5% Triton X-100, 0.3 tablets of cOmplete protease inhibitor cocktail (Roche) and two tablets of Phos-STOP phosphatase inhibitor cocktail (Roche)] using a micropipette tip whose tip has been cut off. The scraped cell-lysis buffer solutions were rotated in a 1.5 ml tube at 4°C for 30 min, centrifuged at 17,300×***g***, 4°C and the supernatant was collected as cell lysate, either stored at −20°C or −80°C, or proceeded on to each experiment.

### Immunoprecipitation

Immunoprecipitation was performed using either Protein G–agarose (Thermo Fisher Scientific) or GFP-Trap beads (Chromotek). For immunoprecipitation using Protein G–agarose, cells were first precleared of nonspecific agarose or protein G binding by rotating with washed Protein G–agarose for 30 min at 4°C. A portion (1%) of the supernatant was collected as the input fraction, and the rest was mixed with washed Protein G–agarose and 1 μl of antibody per 500 μl of cell lysate and rotated for 3 h at 4°C. Agarose beads were then washed with TBS containing cOmplete and Phos-STOP three times and boiled in 1.5× sample buffer for 10 min at 90°C.

For immunoprecipitation using GFP-Trap beads, cell lysates were first diluted with TBS buffer (50 mM Tris-HCl pH 7.6, 150 mM NaCl) to reduce the concentration of Triton X-100. These were next mixed with equilibrated and washed GFP-Trap beads, then rotated for 2 h at 4°C. The samples were then centrifuged at 2500 ***g*** to separate the unbound supernatant from the beads. The beads were washed with TBS buffer for three more times, and were boiled in 2× sample buffer for 10 min at 90°C.

### Isolation of lysosomes

Isolation of lysosomes was conducted as in a previously described protocol (Eguchi et al., 2018). Briefly, cells on a 10 cm dish were cultured in DMEM containing 1 mM HEPES-NaOH (pH 7.2) and 10% Dextran-coated magnetite (DexoMAG 40, Liquids Research, UK) for 24 h, and then chased in normal medium for 24 h. Cells were harvested with trypsin, centrifuged at 60 ***g*** for 5 min, washed with ice-cold PBS, lysed in 2 ml of ice-cold Buffer A [1 mM HEPES, 15 mM KCl, 1.5 mM Mg(Ac)_2_, 1 mM DTT and protease and phosphatase inhibitors) with a Dounce homogenizer, and passed through a 23G needle for eight times. After homogenization, 500 μl of ice-cold Buffer B [220 mM HEPES, 375 mM KCl, 22.5 mM Mg(Ac)_2_, 1 mM DTT, 0.1 mM sucrose, 50 μg/ml DNase I] was immediately added, and samples were inverted five times, incubated for 5 min, then centrifuged at 400 ***g*** for 10 min. The supernatant was then applied to an MS Column (Miltenyi Biotec) set on an OctoMACS separator (Miltenyi Biotec) and equilibrated with 0.5% BSA in PBS, and the flow through was collected. 1 ml of DNase I solution (50 μg/ml DNase I, 0.1 mM sucrose in PBS) was added, and the column was incubated for 10 min and washed with 1 ml of ice-cold sucrose buffer (0.1 mM sucrose in PBS). After removing the column from the OctoMACS separator, lysosomes were eluted with 250 μl of ice-cold sucrose buffer using a plunger.

### *In vitro* kinase assay

Previously purified recombinant Rab29 (Fujimoto et al., 2018) (450 ng) and PKCα or PKCε (Aviva Systems Biology) (50 ng each) were mixed in kinase assay buffer (50 mM Tris-HCl, 10 mM MgCl_2_, 1 mM CaCl_2_, 2 mM DTT, 333 nM PMA and 1 mM ATP) and incubated at 30°C for 30 min on a shaker at 900 rpm. The mixtures were then diluted in 4× sample buffer (4×LDS buffer mixed with 4% (v/v) 2-mercaptoethanol) and boiled at 90°C for 5 min before proceeding on to SDS-PAGE.

### SDS-PAGE and western blotting

For samples that need quantification, BCA assays using a BCA assay kit (TaKaRa) were performed before boiling the lysed cells or mixing medium with a one-third volume of 4× sample buffer for 10-15 min at 90°C. SDS-PAGE was conducted to separate proteins by their size using 7.5%, 10% or 12.5% Tris-glycine gels. Phos-tag SDS-PAGE was performed to separate phosphorylated proteins from non-phosphorylated forms using Phos-tag gels [7.5% Tris-glycine gels supplemented with 150 μM MnCl_2_ and 75 μM Phos-tag acrylamide (Wako)]. For each gel, a lane was kept for molecular mass markers (Precision Plus Dual Color Protein Standard, Bio-Rad). Samples after electrophoresis were transferred to PVDF membranes (Millipore) in blotting buffer (10 or 20% methanol, 25 mM Tris-HCl, 200 mM glycine). Phos-tag gels were rocked in blotting buffer with EDTA to remove excess Mn^2+^ ions. PVDF membranes were then blocked in 5% skim milk in TBS-Tween [Tris-buffered saline (50 mM Tris-HCl pH 7.6, 150 mM NaCl,) mixed with 0.1% Tween 20 (Sigma Aldrich)], or for samples subjected to phospho-antibodies, 5% bovine serum albumin (Sigma-Aldrich) in TBS-Tween, for 30 min at room temperature. Primary antibodies were diluted in Immuno-enhancer (Wako) and incubated with blocked PVDF membranes overnight at 4°C. After subsequent washing with TBS-Tween three times for 5 min each, membranes were incubated with secondary antibodies diluted in Immuno-enhancer for 45 min at room temperature or overnight at 4°C. Then, membranes were washed twice for 10 min each before being immersed in Immunostar reagents (Wako) for chemiluminescence. Chemiluminescent signals were detected by LAS-4000 mini (FUJIFILM) and quantified using Fiji (Schindelin et al., 2012). Uncropped images of immunoblots shown in the figures are provided in Fig. S8.

### Mass spectrometry

For identifying the phosphorylation site of Rab29, the portion of the gel corresponding to phosphorylated Rab29 was cut after Phos-tag SDS-PAGE and digested by either trypsin or a combination of chymotrypsin and elastase using a ProGest robot (DigiLab). The digested samples were then loaded on a nano LC-MS/MS with a Waters NanoAcquity HPLC system interfaced to a ThermoFisher Q Exactive. The retrieved data were then analyzed using a local copy of Mascot (Matrix Science). The procedures after cutting the gel were performed at MS Bioworks LLC (Ann Arbor, MI, USA).

### Quantification of lysosomal size

Lysosomal cross-sectional areas in images obtained from ICC were quantified using Fiji (Schindelin et al., 2012). Image acquisition was performed at *z* levels where lysosomes would be the largest. Lysosomes were traced with an oval tool and their surface areas were obtained by using the measure command. The largest lysosome of all visible cells (only those with GFP–Rab29 expression in Fig. 4) in the images were traced and measured, which was conducted without data anonymization. The surface areas were then recorded in a Microsoft Excel sheet for further analysis.

### Prediction of kinases

The full-length sequence of human Rab29 was analyzed by NetPhos3.1 (Blom et al., 1999).

### Molecular modeling

The structural modeling of proteins was conducted using UCSF ChimeraX (Pettersen et al., 2020).

### Statistical analyses

Statistical analyses were conducted using R platform (The R Foundation; https://www.r-project.org/) or GraphPad Prism 7. For multiple comparisons after ANOVA analysis, Tukey's test was performed when comparing every mean with every other mean, and Dunnett's test was performed when comparing every mean with a control mean. Statistical significance was set to *P*<0.05.

## Supplementary Material

10.1242/joces.261003_sup1 Supplementary information
