# Supplementary material for: Phosphorylation of Rab29 at Ser185 regulates its localization and role in the lysosomal stress response in concert with LRRK2
Source: J Cell Sci. 2023 Jul 21;136(14):jcs261003. doi: 10.1242/jcs.261003 (PMC10399995; doi:10.1242/jcs.261003)
Supplement: Supplementary information [file joces-136-261003-s1.pdf]

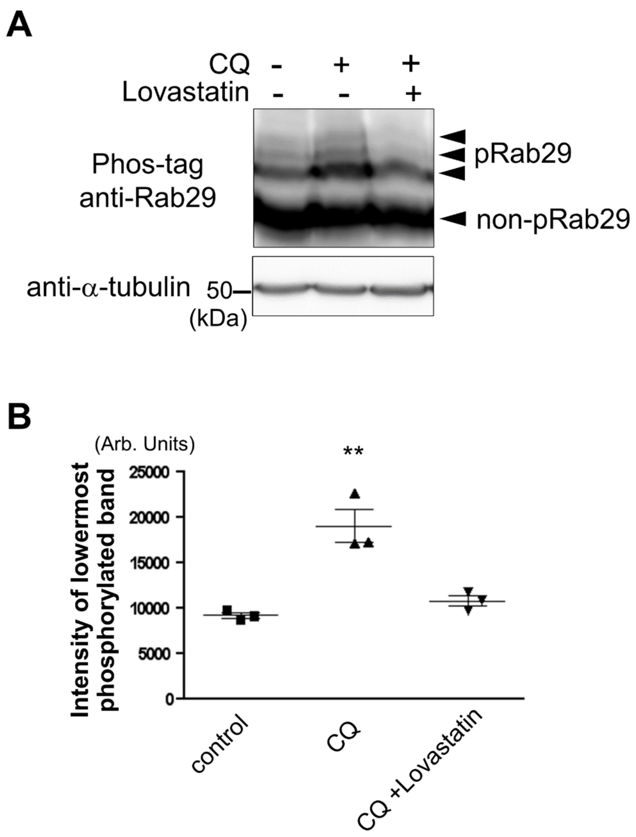

Fig. S1. Rab29 phosphorylation requires a C-terminal prenylation.
(A) Inhibition of CQ-induced Rab29 phosphorylation by lovastatin in HEK293 cells. Representative image of n = 3 trials. **(B)** Quantification of the intensity of the lowermost phosphorylated band in A. One-way ANOVA followed by Dunnett's test against the control, n = 3. **: $p < 0.01$. Error bars indicate s.e.m.

Journal of Cell Science • Supplementary information

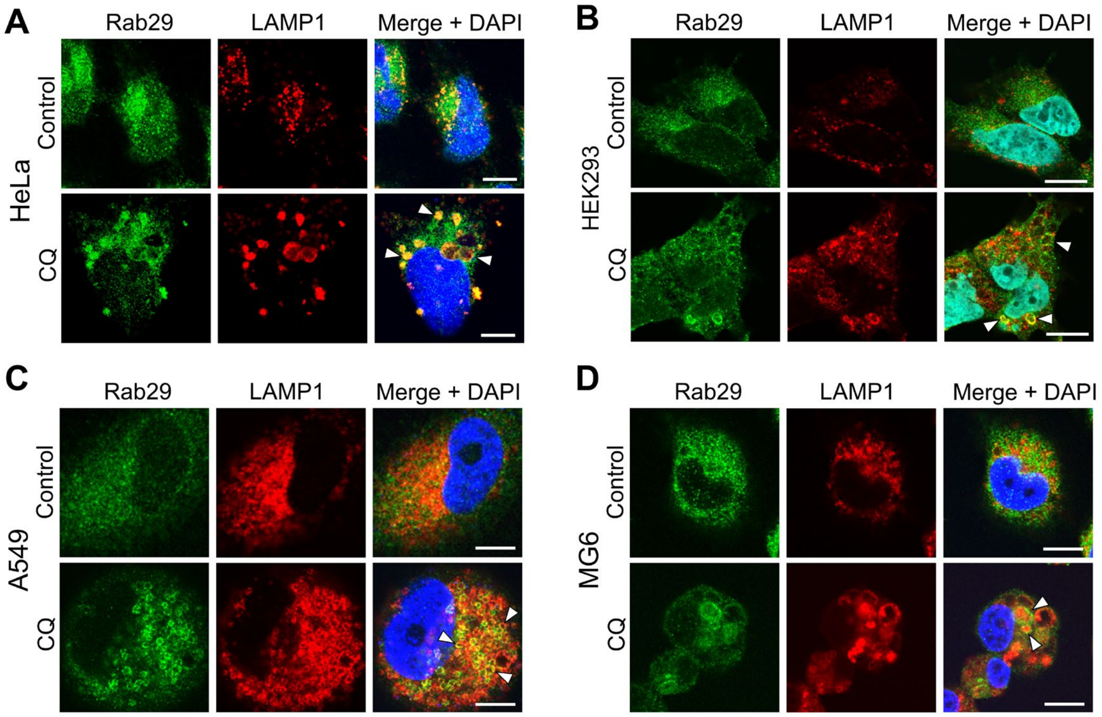

Fig. S2. Endogenous Rab29 localizes to enlarged lysosomes in various cells. Localization of endogenous Rab29 under CQ-treated conditions in **(A)** HeLa, **(B)** HEK293, **(C)** A549, and **(D)** MG6 cells. The arrowheads indicate Rab29 colocalization with LAMP1, a lysosomal marker. Bars = 10 µm.

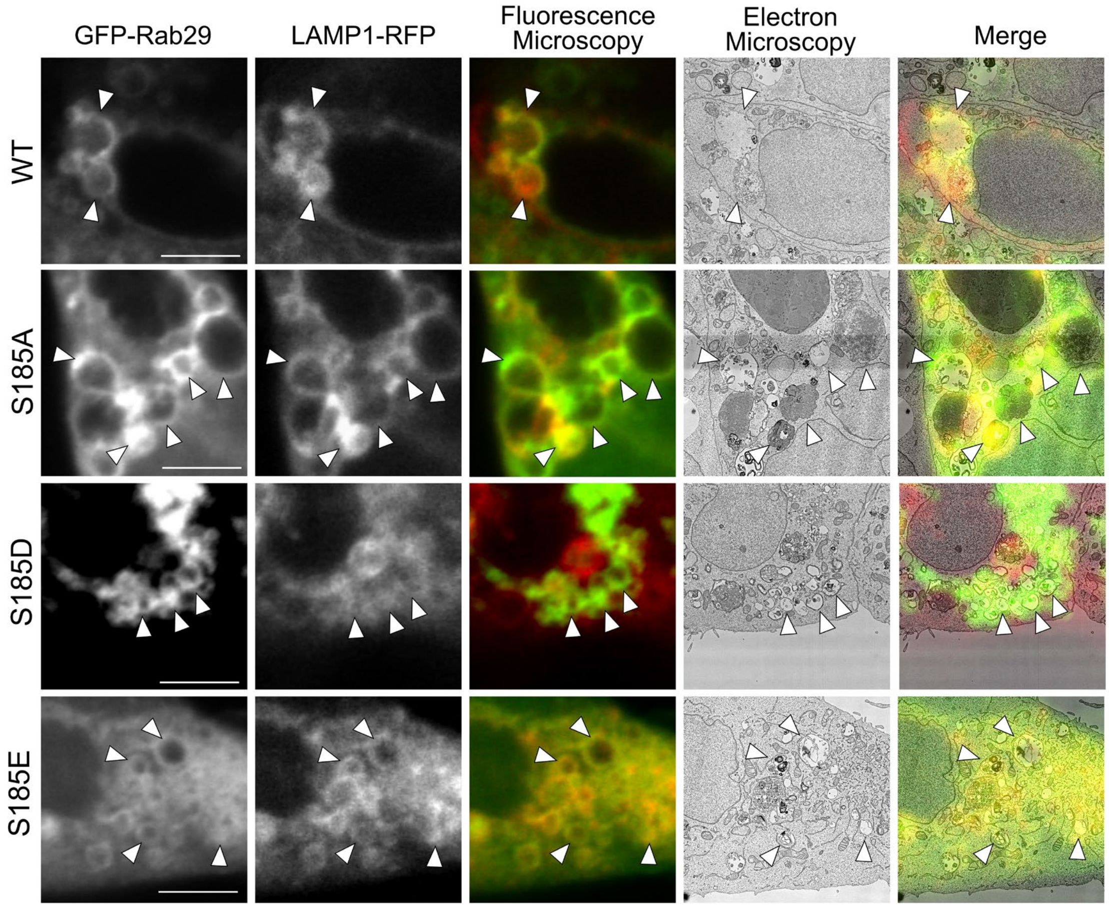

Fig. S3. Correlative light and electron microscopy (CLEM) of Rab29 phospho-site mutants.

CLEM of HEK293 cells overexpressing GFP-Rab29 (mutant described on the left) and LAMP1-RFP, treated with CQ. White arrowheads indicate enlarged lysosomes that are Rab29 positive. Not all lysosomes are marked. Note that the brightness of electron microscopy (EM) images in the CLEM column have been cut down by approximately 1/2 to avoid saturation of the overlapped images. Bars = 5 μm.

Journal of Cell Science • Supplementary information

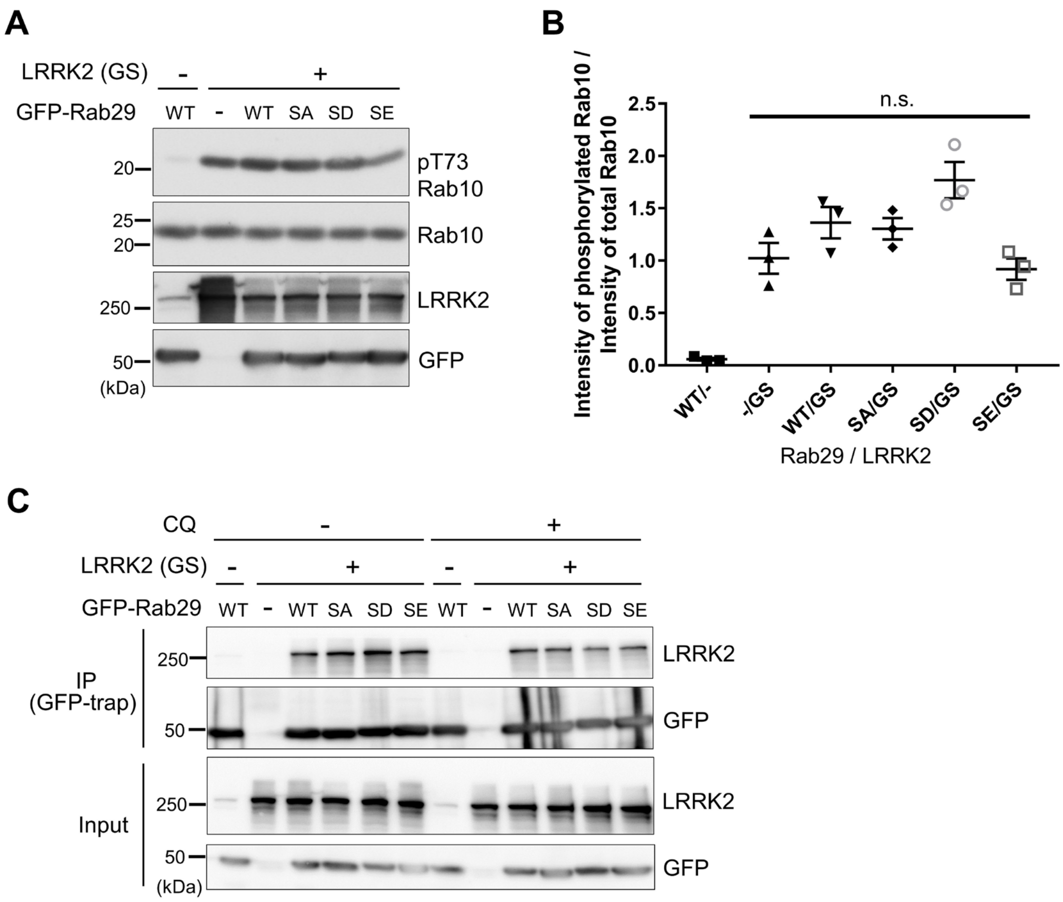

Fig. S4. Rab29 Ser185 phosphomimetics do not alter LRRK2 kinase activity or Rab29-LRRK2 interaction.

(A) Phosphorylation of Rab10 by LRRK2 in HEK293 cells upon overexpression of Rab29 wild-type (WT), S185A mutant (SA) or S185D/E phosphomimetics (SD, SE). GS: G2019S mutant. Representative image of n = 3 trials. **(B)** Quantitative analysis of Rab10 phosphorylation, as shown in A. One-way ANOVA followed by Dunnett's test against control (WT Rab29/ LRRK2 G2019S expression). n.s.: not significant. **(C)** Co-immunoprecipitation of G2019S LRRK2 by GFP-Rab29 in HEK293 cell lysates using an anti-GFP antibody (GFP-trap).

Journal of Cell Science • Supplementary information

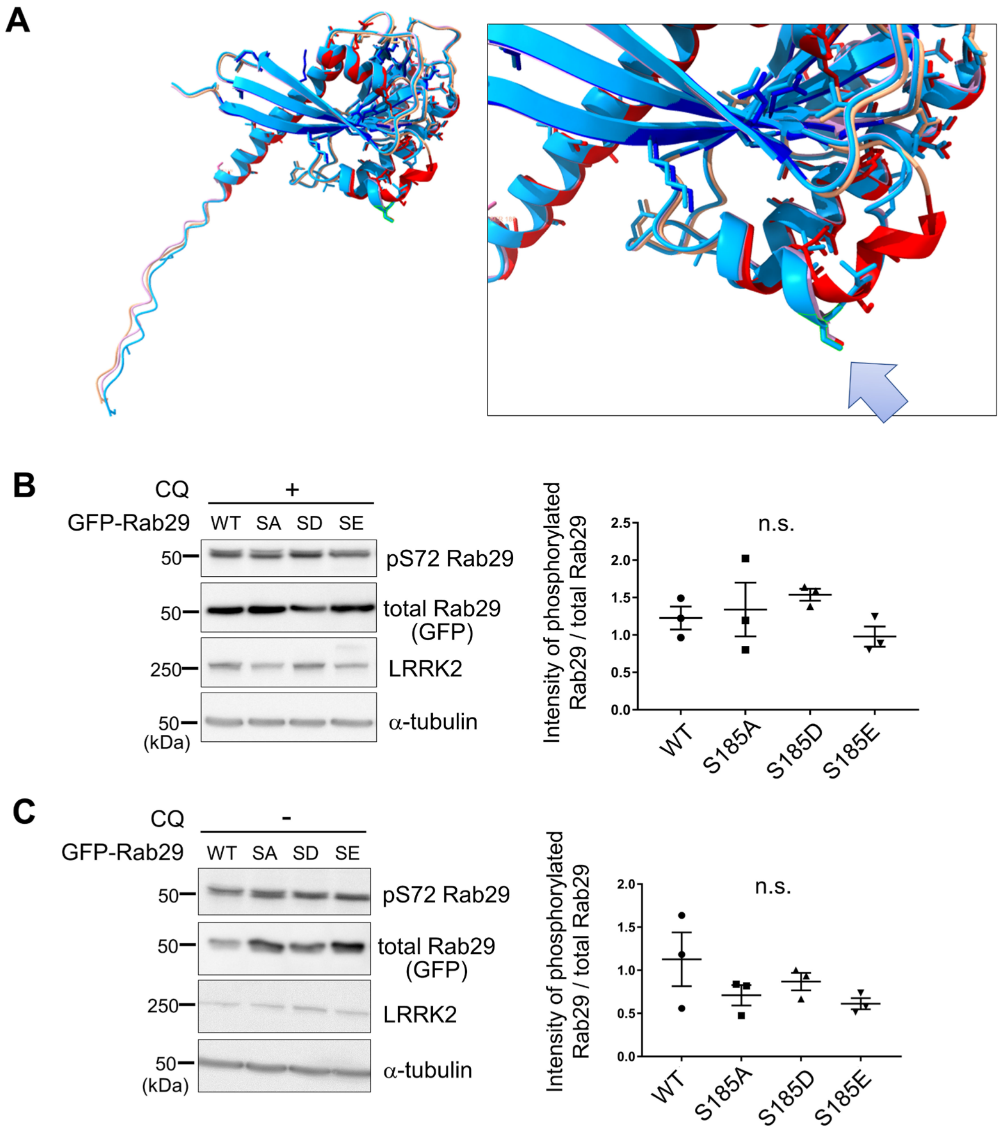

Fig. S5. Rab29 Ser185 phosphomimetics do not alter their phosphorylation by LRRK2 at Ser72.

(A) An AlphaFold2 prediction of S185D and S185E phosphomimetics of Rab29 (colored light blue and pink, respectively) and an inset around the switch II region. The arrow indicates Ser72 residue. **(B)** Phosphorylation of Rab29 at Ser72 in HEK293 cells overexpressing Rab29 S185A mutant (SA) or S185D/E phosphomimetics (SD, SE) upon CQ treatment. The images are representative of n = 3 trials. One-way ANOVA followed by Dunnett's test against WT. n.s.: not significant. **(C)** Phosphorylation of Rab29 at Ser72 in HEK293 cells overexpressing Rab29 S185A mutant (SA) or S185D/E phosphomimetics (SD, SE) without CQ treatment. The images are representative of n = 3 trials. One-way ANOVA followed by Dunnett's test against WT. n.s.: not significant.

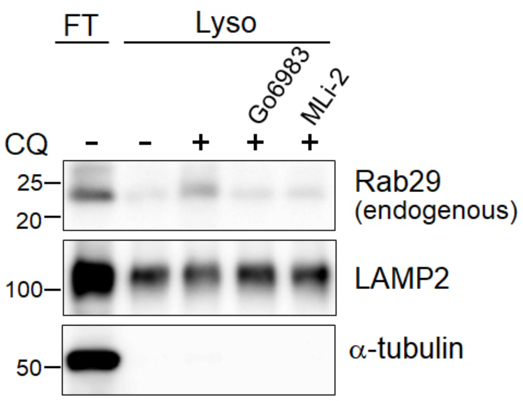

Fig. S6. Inhibition of either PKCs or LRRK2 lower Rab29 localization to lysosomes induced by CQ.

Biochemical analysis of endogenous Rab29 and control proteins in flow through (FT) and lysosomal (Lyso) fractions from HEK293 cells treated with or without CQ and the indicated inhibitors. Representative image of n = 3 trials.

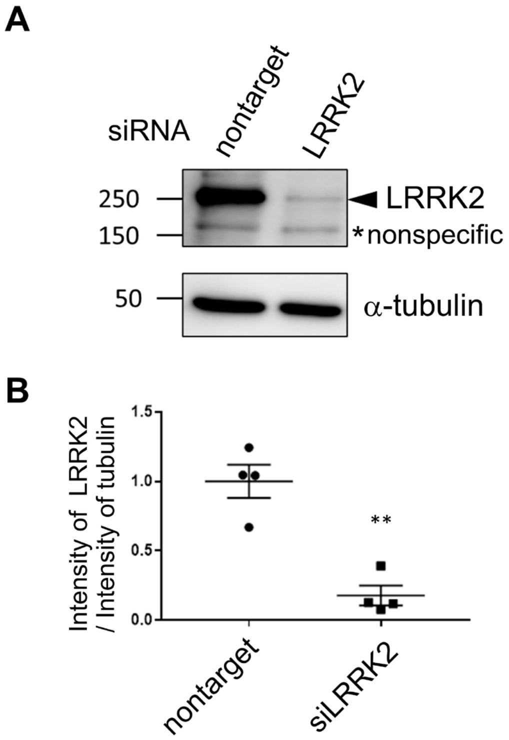

Fig. S7. Confirmation of knockdown of LRRK2 in RAW264.7 cells

(A) Knockdown of LRRK2 protein in RAW264.7 cells by siRNA. Representative image of n = 4 trials. **(B)** Quantification of LRRK2 band intensities in A normalized by those of α-tubulin. Mean of nontarget siRNA-treated samples is set to 1 for easier comparison. n = 4. **: $p < 0.01$ by *t*-test. Error bars indicate s.e.m.

Journal of Cell Science • Supplementary information

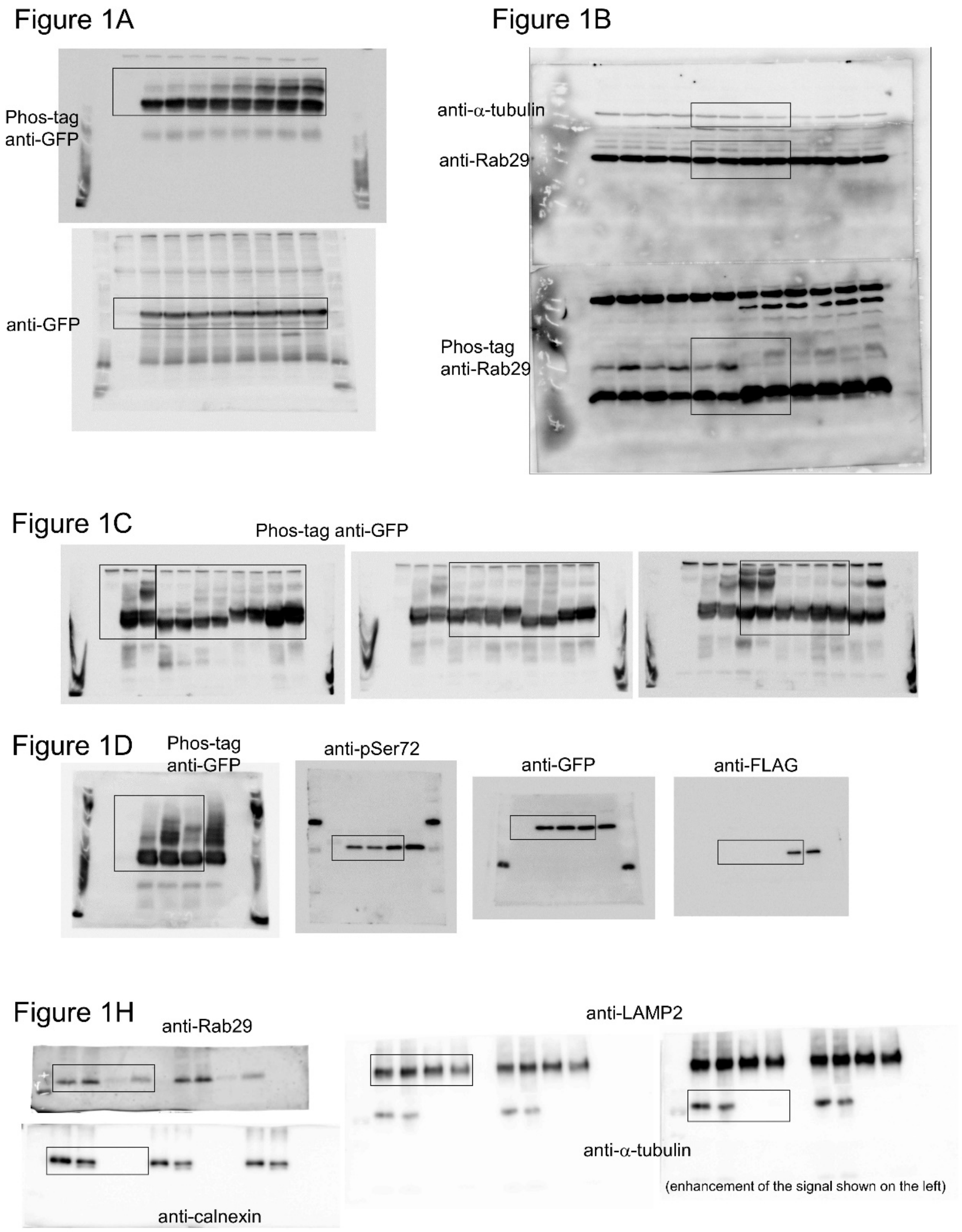

Fig. S8. Blot transparency

Journal of Cell Science • Supplementary information

Figure 2B

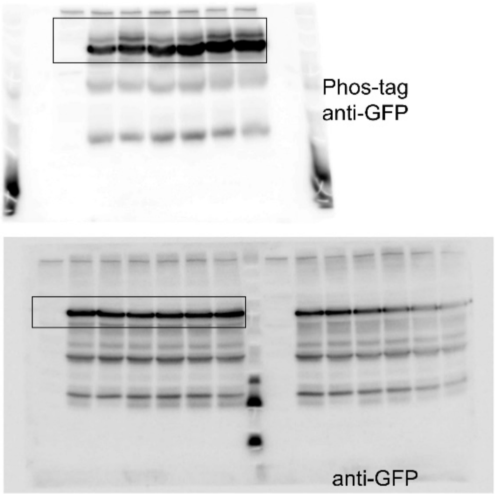

Figure 2E

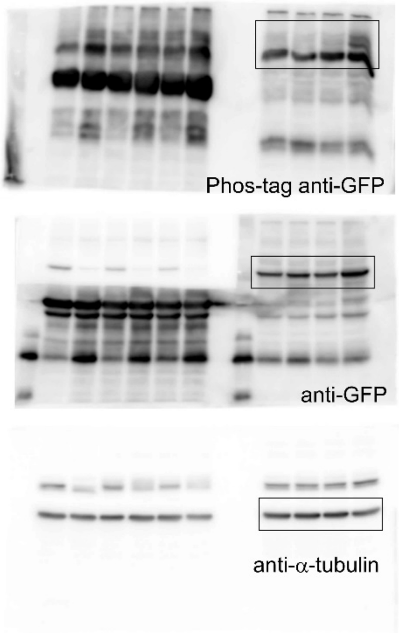

Figure 3C

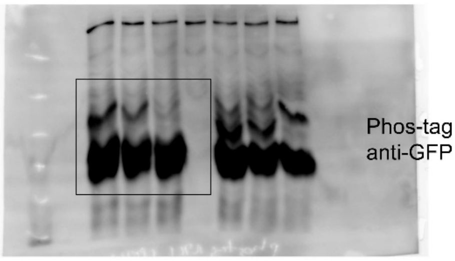

Figure 3D, 3E

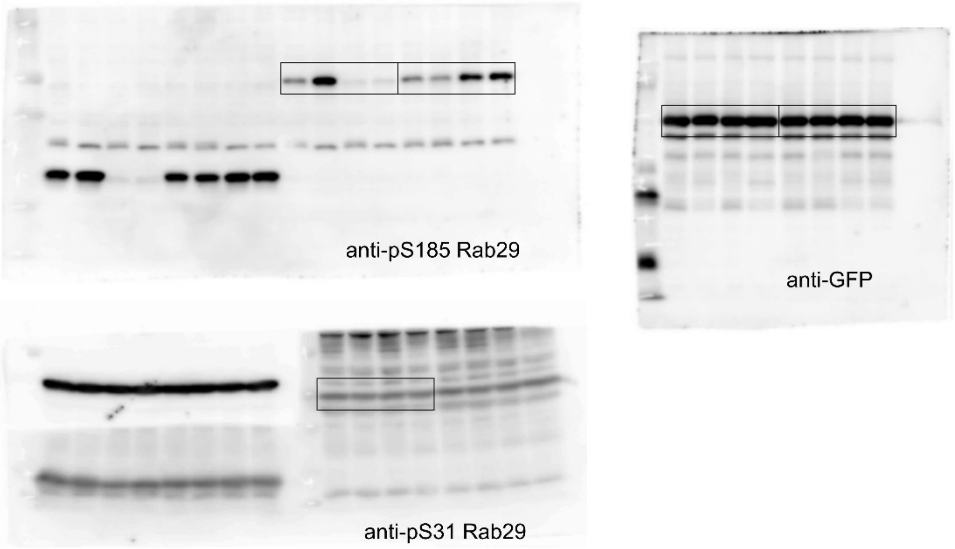

Fig. S8. Blot transparency (continued)

Figure 5A

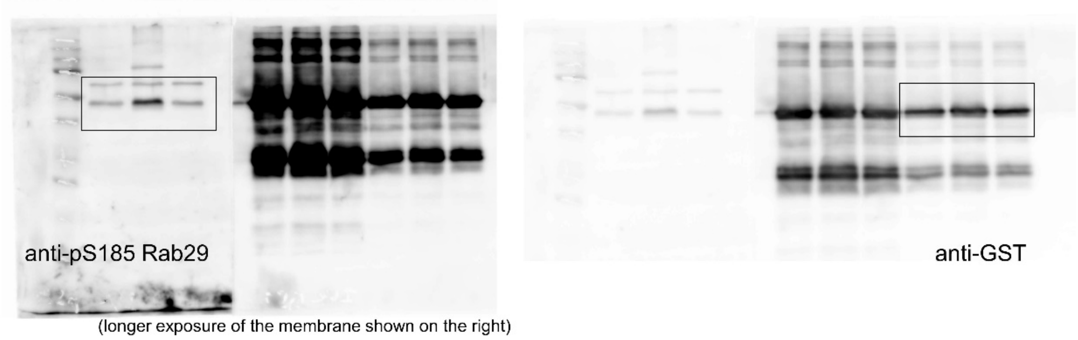

Figure 5B

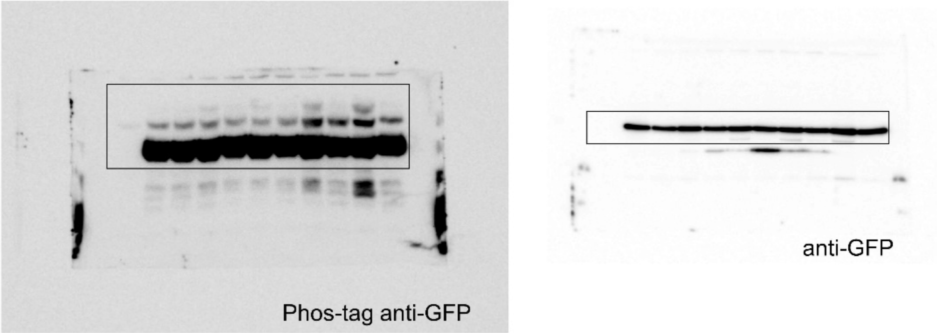

Figure 5C

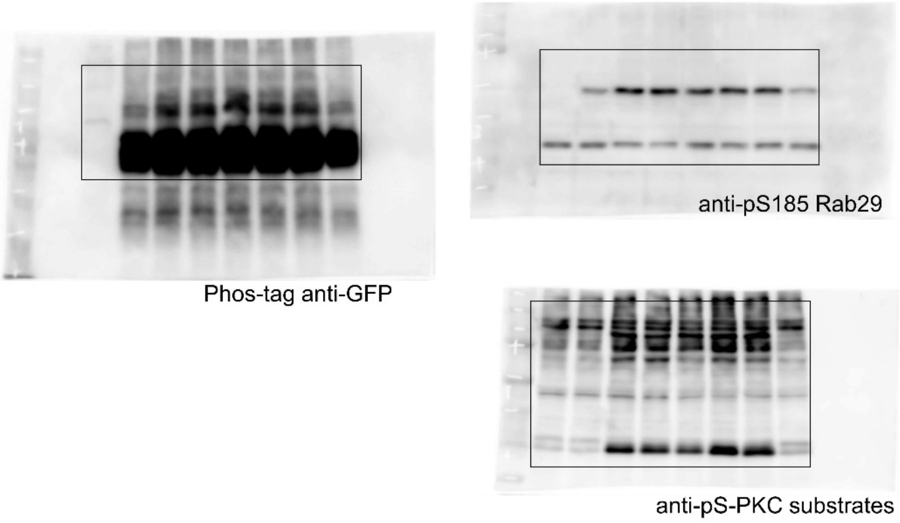

Fig. S8. Blot transparency (continued)

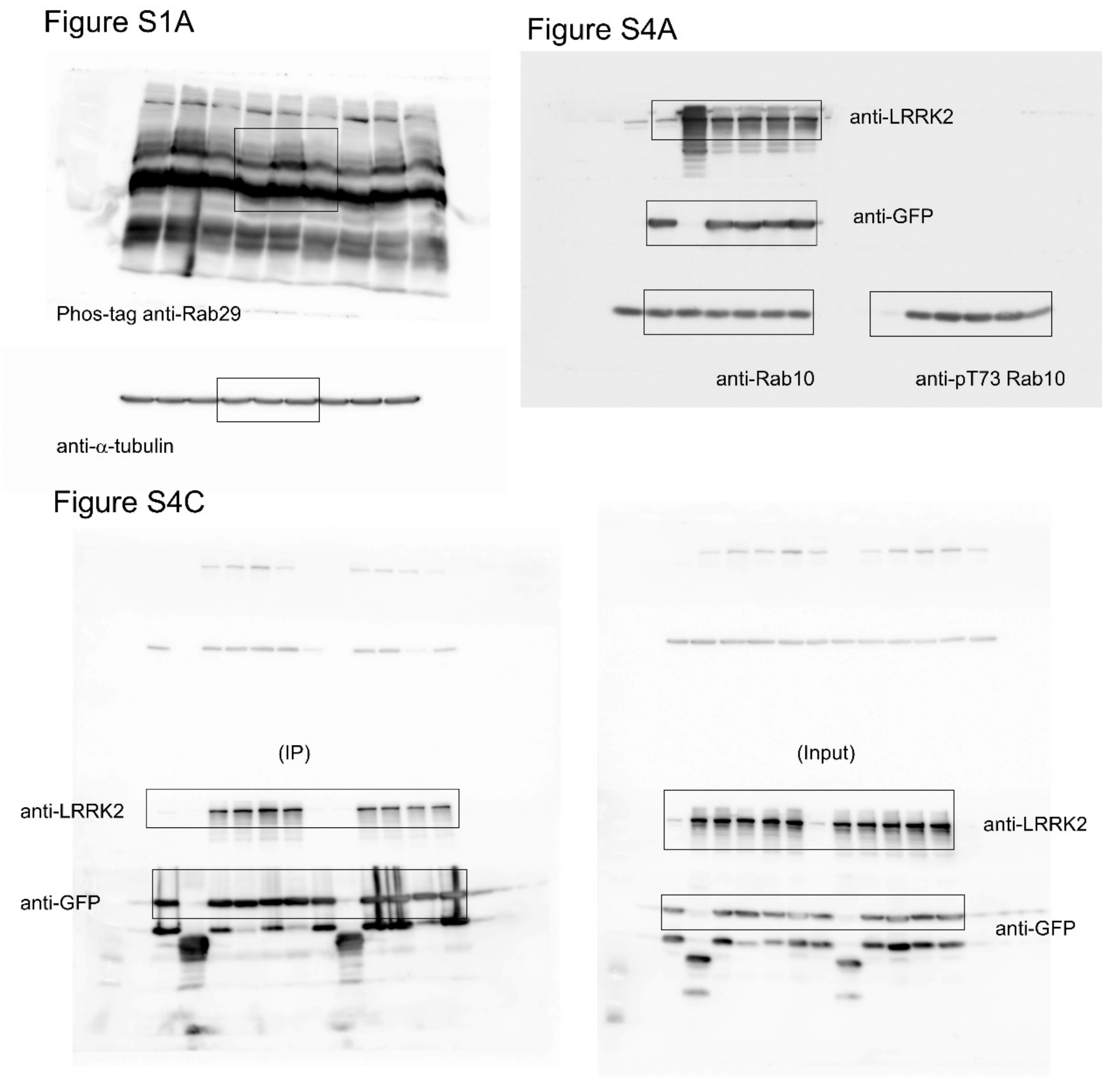

Fig. S8. Blot transparency (continued)

Journal of Cell Science • Supplementary information

Figure S5B, S5C

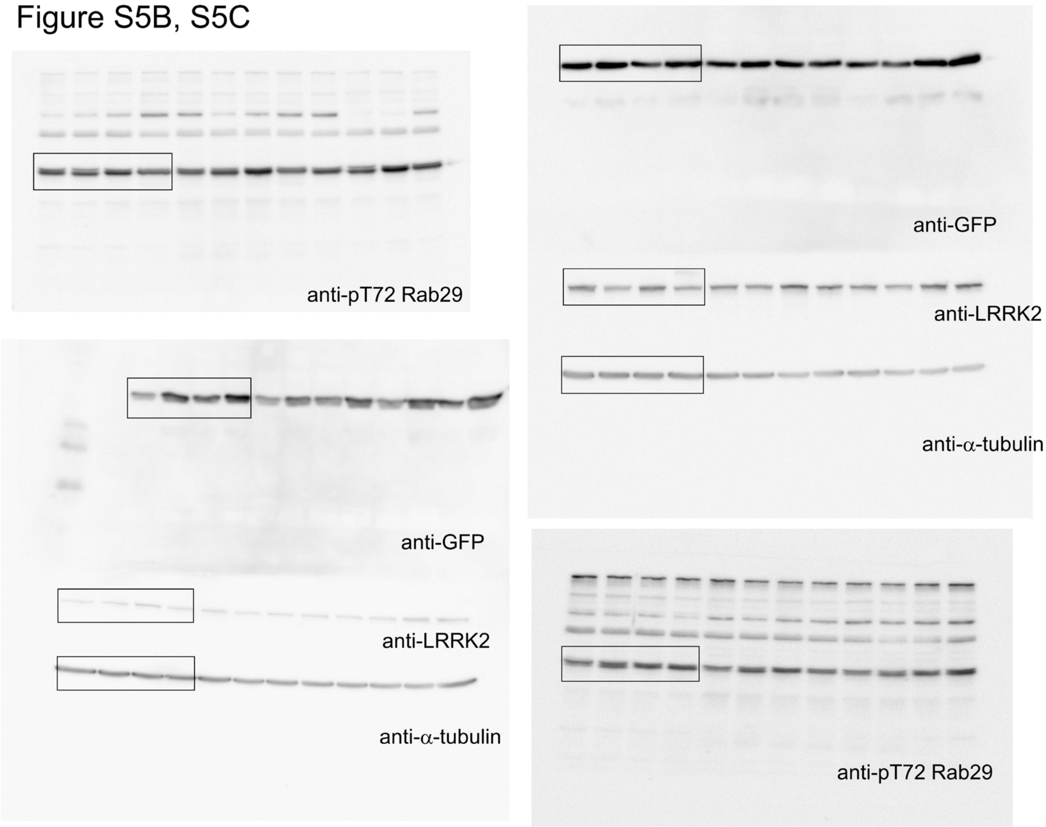

Figure S6

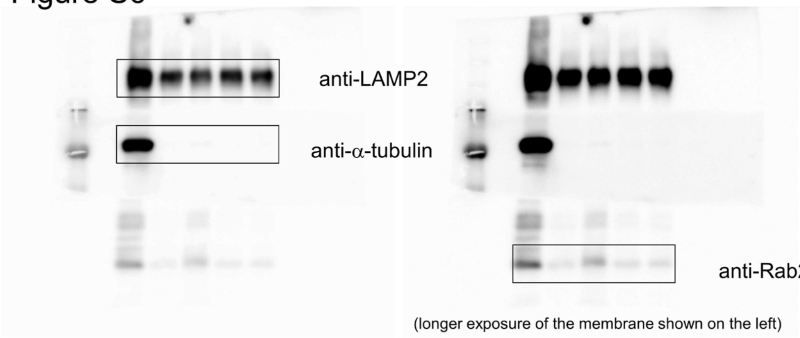

Figure S7

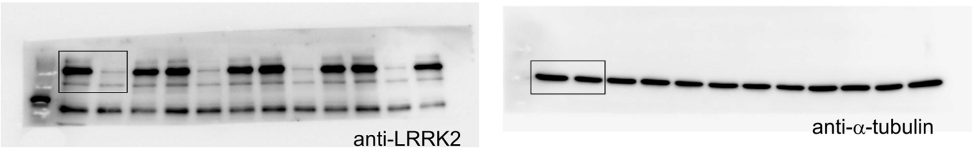

Fig. S8. Blot transparency (continued)

Journal of Cell Science • Supplementary information

Table S1. Prediction of kinases for Rab29 Ser185 by NetPhos 3.1

The full-length sequence of human Rab29 was analyzed by NetPhos3.1 (Blom et al., 1999). The candidate kinases that could phosphorylate Rab29 at Ser185 are shown in order of likelihood. All of them have low scores below 0.5, meaning that they are unlikely to be the responsible kinase for Ser185.

Sequence	#	x	Context	Score	Kinase	Answer
sp_O14966_RAB7L_HUMA	185	S	IMSLSTQGD	0.478	CKII	.
sp_O14966_RAB7L_HUMA	185	S	IMSLSTQGD	0.456	cdc2	.
sp_O14966_RAB7L_HUMA	185	S	IMSLSTQGD	0.45	GSK3	.
sp_O14966_RAB7L_HUMA	185	S	IMSLSTQGD	0.412	CaM-II	.
sp_O14966_RAB7L_HUMA	185	S	IMSLSTQGD	0.368	CKI	.
sp_O14966_RAB7L_HUMA	185	S	IMSLSTQGD	0.355	DNAPK	.
sp_O14966_RAB7L_HUMA	185	S	IMSLSTQGD	0.355	ATM	.
sp_O14966_RAB7L_HUMA	185	S	IMSLSTQGD	0.275	p38MAPK	.
sp_O14966_RAB7L_HUMA	185	S	IMSLSTQGD	0.268	RSK	.
sp_O14966_RAB7L_HUMA	185	S	IMSLSTQGD	0.259	PKG	.
sp_O14966_RAB7L_HUMA	185	S	IMSLSTQGD	0.222	PKA	.
sp_O14966_RAB7L_HUMA	185	S	IMSLSTQGD	0.203	PKC	.
sp_O14966_RAB7L_HUMA	185	S	IMSLSTQGD	0.167	cdk5	.
sp_O14966_RAB7L_HUMA	185	S	IMSLSTQGD	0.085	PKB	.
sp_O14966_RAB7L_HUMA	185	S	IMSLSTQGD	0.075	unsp	.

Journal of Cell Science • Supplementary information
