# Supplementary material for: Phosphorylation of Rab29 at Ser185 regulates its localization and role in the lysosomal stress response in concert with LRRK2
Source: J Cell Sci. 2023 Jul 21;136(14):jcs261003. doi: 10.1242/jcs.261003 (PMC10399995; doi:10.1242/jcs.261003)
Supplement: Supplementary file 1 [file joces-136-261003_review_history.pdf]

Phosphorylation of Rab29 at Ser185 regulates its localization and role in the lysosomal stress response in concert with LRRK2

Tadayuki Komori, Tomoki Kuwahara, Tetta Fujimoto, Maria Sakurai, Ikuko Koyama-Honda, Mitsunori Fukuda and Takeshi Iwatsubo

DOI: 10.1242/jcs.261003

Editor: Michael Way

Review timeline

Submission to Review Commons:	22 September 2022
Submission to Journal of Cell Science:	20 January 2023
Editorial decision:	24 January 2023
First revision received:	18 June 2023
Accepted:	20 June 2023

Reviewer 1

Evidence, reproducibility and clarity

Summary

In this manuscript, Komori et al. examined the role of the LRRK2 substrate and regulator Rab29 in the lysosomal stress response. Briefly, in chloroquine (CQ)-treated HEK293 cells the authors observed an apparent LRRK2-independent increased in Rab29 phosphorylation which was accompanied by translocation of Rab29 to lysosomes. Intriguingly, the authors detected a similar increase in Rab29 phosphorylation when Rab29 was tethered to lysosomes in the absence of CQ treatment. Using mass spectrometry, mutagenesis and a phospho-specific anti-body, the authors mapped the CQ-induced phosphorylation site to S185 and demonstrated its independence from LRRK2. Next, the authors found that PKCa was the kinase responsible for S185 phosphorylation and lysosomal translocation of Rab29. Lastly, the authors showed that in addition to PKCa the lysosomal translocation of Rab29 was also regulated by LRRK2. Overall, Komori and colleagues provide interesting new insights into the phosphorylation-dependent regulation of Rab29. However, there are. Number of technical and conception concerns which should be addressed.

Major points

1. Figure 1F: the localization of Rab29 to lysosomes is not convincing at all. The authors should either provide more representative image examples or image the cells at a higher resolution. The authors should also confirm the CQ-induced lysosomal localization of Rab29 in a different cell type (e.g., HEK293). Moreover, the authors should show that prenylation of Rab29 is required for its CQ-induced phosphorylation.
2. The rapalog-induced increase in Rab29 phosphorylation in Figure 2D is not convincing since there is at least 2-3-fold more Rab29 in FRB-LAMP1 expressing cells compared to their FRB-FIS1 counterparts. An independent loading control is also missing. This is a key experiment and should be properly controlled and quantified. In addition, can CQ treatment drive 2xFKBP GFP-Rab29 from mitochondria to lysosomes (in the presence of rapalog and FRB-Fis1)?
3. Figure 4A-C: Are these stable Rab29 expressing cells? If not, the quantification of "the size of largest lysosome in EACH cell" becomes very problematic. This analysis should be repeated with stable Rab29 variant cells in a background lacking endogenous Rab29. Furthermore, the LAMP1 signal is too dim to see any convincing colocalization (e.g., with WT) or the lack thereof (e.g., in the case of S185D). Lastly, the authors should corroborate their findings with an ultrastructural analysis since the electron microscopy would definitively be more suitable for this type of

© 2023. Published by The Company of Biologists under the terms of the Creative Commons Attribution License (https://creativecommons.org/licenses/by/4.0/).

measurements.
4. The lysosomal colocalization of Rab29 in Figure 5C is again not convincing. This analysis needs to be repeated with high resolution imaging. Along similar lines: why not all cells in Figure 5E and Figure 5G show Rab29- and LRRK2-positive structures? How do the authors know which of these phenotypes is the prevalent one?
5. The authors need to show the level of LRRK2 depletion (Figure 6). Given the role of LRRK2 in driving lysosomal Rab29 translocation, the importance of the LRRK2 independent pS185 for this process remains unclear.
6. In general, the authors employ an alternative, biochemical assay (e.g., LysoIP) for the lysosomal translocation of Rab29. This would in particular help to clarify the effect of the Rab29 variants and LRRK2 inhibition.

Minor points

7. It is not clear what the authors mean by "lysosomal overload stress". Since mature lysosomal incoming pathways such as autophagy or endocytosis are disrupted by CQ, it is difficult to picture an overload. Maybe rephrasing would help to clarify this.
8. Which cell type is used for the IF analysis in Figure 2C? This information is in general quite sparse. The authors should clearly state the cell type for each experiment/Figure.
9. Figure 2C is lacking the control IF staining for mitochondria (to which 2xFKBP-GFP-Rab29 is assumed be recruited upon co-expression with FRB-FIS1).
10. Molecular weight markes should be provided for all immunoblot experiments.

Significance

Please see above.

Reviewer 2

Evidence, reproducibility and clarity

The data in the manuscript convincingly demonstrates that lysosomal overload by Chloroquine treatment induces Rab29 localisation to the lysosomes and that this membrane association is dependent on PKCalpha-dependent phosphorylation at Ser185.
We have a number of rather minor comments listed below: Figure 1
- Are the images in figure 1F representative? i.e. does Rab29 always colocalise to such enlarged lysosomes upon CQ treatment and does CQ treatment always drastically alter the cellular distribution of Rab29?
- The use of the quantification ratio of cells with Rab29-positive lysosomes in figure 1G might be slightly misleading as it does not allow the reader to understand to what extent Rab29 localisation at lysosomes upon CQ treatment. We recommend using a simpler quantification, such as by measuring the average colocalisation of Rab29 and LAMP1 per cell.

Figure 2

- Considering that the "forced localisation technique" induces a non-physiological colocalization of non-endogenous Rab29 to lysosomes, it may be an overestimation to conclude just from these data that phosphorylation of Rab29 occurs on the lysosomal surface. This is also quite in contrast with the later finding that phosphorylation by PKCalpha promotes lysosome localization of Rab29. It seems more reasonable to conclude that Rab29 can be phosphorylated when localised at the lysosomes (as opposed to other organelles such as mitochondria). If the authors feel strongly about this point they might need to find a less non-physiological assay.
- The increasing levels of non-phosphorylated Rab29 over the indicated time course of AP21967 treatment in Figure 2B are concerning. First, could you provide an explanation for this clear increase in both non-p-Rab29 and p-Rab29 in the phostag but not the normal gel? Second, could all quantifications of p-Rab29 be made relative to the non-p-Rab29?

© 2023. Published by The Company of Biologists under the terms of the Creative Commons Attribution License (https://creativecommons.org/licenses/by/4.0/).

Figure 3
- It is not clear how the Rab29 phosphomimetics are acting as dominant active preventing lysosomal enlargement. Authors should speculate or repeat the experiments in absence of endogenous Rab29 to clarify the matter.

- Overall, there is some missing information regarding repeats for Western blots, such as those in figure 3C, 3D and 3E. Please add indications about repeats in the figure legend or methods.

Figure 4

- We recommend using a more transparent and simple quantification method, such as average size of lysosomes per cell.

Figure 5

- To further demonstrate that PKCalpha phosphorylates endogenous Rab29 at Ser185, we recommend reperforming the Go3983/PMA treatment in figure B with the anti-p-Ser185 antibody. It may be sufficient to perform the treatment only at 4 or 8 hours, simply to provide stronger evidence regarding the phosphorylation of endogenous Rab29.
- It is not clear whether the activity of PMA in the assay is due to inhibition of PKCalpha. Are the effects ablated by PKCalpha KD
- For figure 5D and 5F, As with figure 1G, we recommend using a more straightforward and impartial method of quantification such as simply measuring the colocalisation of Rab29 with LAMP1.

Figure 6

- Again, we recommend altering the methods of quantification

Figure 7

- The model in figure 7 however seems to suggest that Rab29 associates to lysosomal membranes independently, and is then stabilised at the membranes by LRRK2 and PKCalpha - a point which is not directly supported by the data.

English proofreading should be improved: "CQ was treated to HEK293" (page 4), "As we assumed that this phosphorylation is independent of LRRK2" as an opening line (page 5),

Significance

These cell biology findings are important in the field as both Rab29 and LRRK2 are implicated in the pathogenesis of Parkinson disease. The phosphorilation of Ser185 of Rab29 by PKCalpha is novel and contributes to our understanding of Rab29 and LKRR2 regulation. One limitation of the study is that is conducted in only two cell types quite unrelated to the disease, so how general and disease relevant are the findings it is not clear. Most of the data are solid. There are two experiments whose results are difficult to interpret and a few controls missing. Also a few issues with quantifications, all of which is described in details above and will need to be fixed prior to publication. My expertise for this paper is in the cell biology of lysosomal function.

Author response to reviewers' comments

1. General Statements [optional]

We would like to thank the reviewers for taking time in reviewing and commenting on our paper. The comments were very constructive and conscientious, thanks to their expertise in the field.

© 2023. Published by The Company of Biologists under the terms of the Creative Commons Attribution License (https://creativecommons.org/licenses/by/4.0/).

These comments and the revisions would surely make this paper a better and more robust finding in the field.

The comments were about clearer explanations, increasing the quality of the data and additional experiments for a stronger conclusion, all of which we are eager to accomplish. Now we have sorted out the problems and planned the experiments required in the revision, as detailed below.

2. Description of the planned revisions

Reviewer #1 (Evidence, reproducibility and clarity (Required)):
Summary
In this manuscript, Komori et al. examined the role of the LRRK2 substrate and regulator Rab29 in the lysosomal stress response. Briefly, in chloroquine (CQ)-treated HEK293 cells the authors observed an apparent LRRK2-independent increased in Rab29 phosphorylation which was accompanied by translocation of Rab29 to lysosomes. Intriguingly, the authors detected a similar increase in Rab29 phosphorylation when Rab29 was tethered to lysosomes in the absence of CQ treatment. Using mass spectrometry, mutagenesis and a phospho-specific anti- body, the authors mapped the CQ-induced phosphorylation site to S185 and demonstrated its independence from LRRK2. Next, the authors found that PKCa was the kinase responsible for S185 phosphorylation and lysosomal translocation of Rab29. Lastly, the authors showed that in addition to PKCa the lysosomal translocation of Rab29 was also regulated by LRRK2. Overall, Komori and colleagues provide interesting new insights into the phosphorylation-dependent regulation of Rab29. However, there are. Number of technical and conception concerns which should be addressed.

Major points
1) Figure 1F: the localization of Rab29 to lysosomes is not convincing at all. The authors should either provide more representative image examples or image the cells at a higher resolution. The authors should also confirm the CQ-induced lysosomal localization of Rab29 in a different cell type (e.g., HEK293).

-- We will replace Fig 1F pictures with slightly more magnified images with higher resolution. We will also include additional cell types (HEK293, and other cells that are predicted to express endogenous Rab29); Reviewer #2 also raised this point (see Reviewer #2 comment on Significance).

Moreover, the authors should show that prenylation of Rab29 is required for its CQ-induced phosphorylation.

-- We will test the effect of lovastatin, a HMG-CoA reductase inhibitor that causes the depletion of the prenylation precursor geranylgeranyl diphosphate from cells (Binnington et al., *Glycobiology* 2016, Gomez et al, *J Cell Biol* 2019), or 3-PEHPC, a GGTase II specific inhibitor that also causes the inhibition of Rab prenylation (Coxon FP et al, *Bone* 2005).

2) The rapalog-induced increase in Rab29 phosphorylation in Figure 2D is not convincing since there is at least 2-3-fold more Rab29 in FRB-LAMP1 expressing cells compared to their FRB- FIS1 counterparts. An independent loading control is also missing. This is a key experiment and should be properly controlled and quantified. In addition, can CQ treatment drive 2xFKBP GFP- Rab29 from mitochondria to lysosomes (in the presence of rapalog and FRB-Fis1)?

-- We will carefully examine another round of rapalog-induced phosphorylation of Rab29, with an independent loading control such as alpha-tubulin. The immunoblot analysis will be made against the intensity of non-p-Rab29. The response to the latter question was described in the section 4 below.

3) Figure 4A-C: Are these stable Rab29 expressing cells? If not, the quantification of "the size of largest lysosome in EACH cell" becomes very problematic. This analysis should be repeated with stable Rab29 variant cells in a background lacking endogenous Rab29. Furthermore, the LAMP1 signal is too dim to see any convincing colocalization (e.g., with WT) or the lack thereof (e.g., in the case of S185D).

-- The cells shown in Figure 4 are HEK293 cells transiently expressing Rab29, and the issue of

© 2023. Published by The Company of Biologists under the terms of the Creative Commons Attribution License (https://creativecommons.org/licenses/by/4.0/).

quantification was described in the section 3 below. We agree that the signal of LAMP1 was dim, and it turned out that the confocal microscope we used had problems with the sensitivity of the red channels. We will be taking another round of these images with a new confocal microscope.

Lastly, the authors should corroborate their findings with an ultrastructural analysis since the electron microscopy would definitively be more suitable for this type of measurements.

-- We are planning to obtain electron microscopic images, according to this reviewer's request. We plan to invite an expert in electron microscopy analysis as a co-author.

4) The lysosomal colocalization of Rab29 in Figure 5C is again not convincing. This analysis needs to be repeated with high resolution imaging.

-- Again, we will repeat this experiment with a new confocal microscope, with the hope that it would yield better images.

5) The authors need to show the level of LRRK2 depletion (Figure 6). Given the role of LRRK2 in driving lysosomal Rab29 translocation, the importance of the LRRK2 independent pS185 for this process remains unclear.

-- We will add the level of LRRK2 on its knockdown; we have experienced that LRRK2 knockdown usually occurs with more than 50% efficiency every time. The response to the latter comment was described in the section 3 below.

6) In general, the authors employ an alternative, biochemical assay (e.g., LysoIP) for the lysosomal translocation of Rab29. This would in particular help to clarify the effect of the Rab29 variants and LRRK2 inhibition.

-- We have previously shown that the overexpressed Rab29 (and LRRK2) is enriched in the lysosomal fraction from CQ-treated cells, which was performed using dextran-coated magnetite (Eguchi et al, *PNAS* 2018). Using the same biochemical method, we will show the enrichment of endogenous Rab29 in the lysosomal fraction.

Minor points
9) Figure 2C is lacking the control IF staining for mitochondria (to which 2xFKBP-GFP-Rab29 is assumed be recruited upon co-expression with FRB-FIS1).

-- We will stain the cells with MitoTracker to ensure that anchoring away of 2xFKBP-GFP-Rab29 by FRB-Fis1 results in mitochondrial localization.

Reviewer #2 (Evidence, reproducibility and clarity (Required)):

The data in the manuscript convincingly demonstrates that lysosomal overload by Chloroquine treatment induces Rab29 localisation to the lysosomes and that this membrane association is dependent on PKCalpha-dependent phosphorylation at Ser185.
We have a number of rather minor comments listed below: Figure 2
• The increasing levels of non-phosphorylated Rab29 over the indicated time course of AP21967 treatment in Figure 2B are concerning. First, could you provide an explanation for this clear increase in both non-p-Rab29 and p-Rab29 in the phostag but not the normal gel? Second, could all quantifications of p-Rab29 be made relative to the non-p-Rab29?
-- We will try another round of rapalog-induced phosphorylation of Rab29, with an independent loading control. The immunoblot analysis will be made against the intensity of non-p-Rab29. Reviewer #1 raised a similar concern on Figure 2D.

Figure 5
• To further demonstrate that PKCalpha phosphorylates endogenous Rab29 at Ser185, we recommend reperforming the Go3983/PMA treatment in figure B with the anti-p-Ser185 antibody. It may be sufficient to perform the treatment only at 4 or 8 hours, simply to provide stronger evidence regarding the phosphorylation of endogenous Rab29.

© 2023. Published by The Company of Biologists under the terms of the Creative Commons Attribution License (https://creativecommons.org/licenses/by/4.0/).

-- We will give a try, although the anti-phosphorylated protein antibodies that we tried never worked for phos-tag SDS-PAGE. With the conventional western blot, we will be able to try this experiment.

• It is not clear whether the activity of PMA in the assay is due to inhibition of PKCalpha. Are the effects ablated by PKCalpha KD

-- We will test the knockdown of PKCalpha, beta, gamma and delta by siRNAs to further narrow down the effects of PKC-dependent phosphorylation of Rab29.

Reviewer #2 (Significance (Required)):
These cell biology findings are important in the field as both Rab29 and LRRK2 are implicated in the pathogenesis of Parkinson disease. The phosphorilation of Ser185 of Rab29 by PKCalpha is novel and contributes to our understanding of Rab29 and LKRR2 regulation. One limitation of the study is that is conducted in only two cell types quite unrelated to the disease, so how general and disease relevant are the findings it is not clear. Most of the data are solid. There are two experiments whose results are difficult to interpret and a few controls missing. Also a few issues with quantifications, all of which is described in details above and will need to be fixed prior to publication. My expertise for this paper is in the cell biology of lysosomal function.

-- The issue that only two cell types were analyzed was also raised by reviewer #1, so we will examine additional cell types, especially those that are predicted to express endogenous Rab29. Our responses to other issues raised are described elsewhere. Thank you for these insightful comments.

3. Description of the revisions that have already been incorporated in the transferred manuscript

Figure 4A-C: Are these stable Rab29 expressing cells? If not, the quantification of "the size of largest lysosome in EACH cell" becomes very problematic. This analysis should be repeated with stable Rab29 variant cells in a background lacking endogenous Rab29. (Reviewer #1)

-- As described in the section 2 above, the cells shown in Figure 4 are HEK293 cells transiently expressing Rab29. We are sorry that the description “the size of largest lysosome in each cell” was misleading. As we analyzed only cells overexpressing GFP-Rab29 that were marked with GFP fluorescence, we believe that transient expression should not be a problem. To avoid any misunderstandings, we have described in Figure 4 legends that only lysosomes in Rab29- positive cells (and all cells expressing Rab29) were included in the analysis of the largest lysosome of each cell.

Regarding the effect of endogenous Rab29 in Figure 4 experiments, Reviewer #2 similarly raised the issue on whether Rab29 phosphomimetics are acting as dominant active, preventing lysosomal enlargement. On this point, we have previously reported that knockdown of endogenous Rab29 causes the enhancement of lysosomal enlargement upon CQ treatment (Figure 5I,J of Eguchi et al, *PNAS* 2018), suggesting that the lysosome-deflating effect by phosphomimetics is a dominant active effect rather than dominant negative suppressing endogenous Rab29. This point is considered significant, and thus has been explained in the results section (page 7, lines 168-171).

Along similar lines: why not all cells in Figure 5E and Figure 5G show Rab29- and LRRK2- positive structures? How do the authors know which of these phenotypes is the prevalent one? (Reviewer #1)

-- As for the ratio of cells with Rab29- and LRRK2-positive structures, it seems reasonable given that different cells have different levels of exposure to lysosomal stress and that the response is transient and does not occur simultaneously. The ratio of these positive cells may also vary depending on the cell culture conditions. Since Rab29- and LRRK2-positive structures are rarely seen in control cells, we think this would be a meaningful phenotype even if only 20-30% of cells show such structures. The result that the ratio of localization changes is not 100% is now noted in the results section explaining Figure 1G (page 4-5, lines 108-110) where the immunocytochemical

© 2023. Published by The Company of Biologists under the terms of the Creative Commons Attribution License (https://creativecommons.org/licenses/by/4.0/).

data first appears.

Given the role of LRRK2 in driving lysosomal Rab29 translocation, the importance of the LRRK2 independent pS185 for this process remains unclear. (Reviewer #1)

-- Our data suggested that Rab29 is stabilized on lysosomes only when LRRK2-mediated phosphorylation and S185 phosphorylation both occur on Rab29 molecule (as shown in Figure 7 scheme), so we believe there is no contradiction. We have now described more clearly about this notion at the end of the results section (page 9, lines 235-236).

It is not clear what the authors mean by "lysosomal overload stress". Since mature lysosomal incoming pathways such as autophagy or endocytosis are disrupted by CQ, it is difficult to picture an overload. Maybe rephrasing would help to clarify this. (Reviewer #1)

-- Chloroquine (CQ) is known as a lysosomotropic agent that accumulates within acidic organelles due to its cationic and amphiphilic nature, causing lysosome overload and osmotic pressure elevation, and this is what we call “lysosomal overload stress”. The well-known effects of CQ to disrupt lysosomal incoming pathways are ultimately caused by the above consequences. Also, we have previously reported that lysosomal recruitment of LRRK2 is caused by CQ but not by bafilomycin A1, the latter being an inducer of lysosomal pH elevation, or by vacuolin-1 that enlarges lysosomes without inducing lysosomal overload/pH elevation (Eguchi et al, *PNAS* 2018), and further found that not only CQ but also other lysosomotropic agents commonly induced LRRK2 recruitment (Kuwahara et al, *Neurobiol Dis* 2020). We thus have described the effect of CQ as “overload”. However, it is true that we have not provided a clear explanation for readers, so we have added some notes for lysosomal overload stress in the introduction section (page 3, lines 69-71)

Which cell type is used for the IF analysis in Figure 2C? This information is in general quite sparse. The authors should clearly state the cell type for each experiment/Figure. (Reviewer #1)

-- We have added cell type information that was missing in several places in the manuscript. We are very sorry for the inconveniences. For clarification, HEK293 cells were used in Figure 2C.

Are the images in figure 1F representative? i.e. does Rab29 always colocalise to such enlarged lysosomes upon CQ treatment and does CQ treatment always drastically alter the cellular distribution of Rab29? (Reviewer #2)

-- The images in Figure 1F are representative of when Rab29 is recruited, but it is not seen in all cells, and the ratio of recruitment (~80%) is shown in Figure 1G. Reviewer #1 also asked why Rab29 recruitment is not seen in all cells, and we gave the same answer above. It may be reasonable to speculate that different cells have different levels of exposure to lysosomal stress and that the response is transient and does not occur simultaneously. The ratio of these positive cells may also vary depending on the cell culture conditions. For the readers’ clarity, we have added that the ratio of localization change of Rab29 is not 100% and is comparable to that of LRRK2 previously reported (page 4-5, lines 108-110).

Considering that the "forced localisation technique" induces a non-physiological colocalization of non-endogenous Rab29 to lysosomes, it may be an overestimation to conclude just from these data that phosphorylation of Rab29 occurs on the lysosomal surface. This is also quite in contrast with the later finding that phosphorylation by PKCalpha promotes lysosome localization of Rab29. It seems more reasonable to conclude that Rab29 can be phosphorylated when localised at the lysosomes (as opposed to other organelles such as mitochondria). If the authors feel strongly about this point they might need to find a less non-physiological assay. (Reviewer #2)

-- Yes, it could be an overestimation, and as we do not have better means to conduct a less non-physiological assay, we have modified the description from “occurred on the lysosomal surface” to “could occur on the lysosomal surface” (page 5, line 112 (subtitle) and line 128).

Regarding the comparison with the later finding that phosphorylation by PKCalpha promotes lysosome localization of Rab29, these data (Figure 2 and 5) could be explained with a single

© 2023. Published by The Company of Biologists under the terms of the Creative Commons Attribution License (https://creativecommons.org/licenses/by/4.0/).

speculation: phosphorylation of Rab29 on lysosomal membranes could retain Rab29 on the membranes for a longer time. It is not easy to decipher which comes first, association with membranes or phosphorylation of Rab29, in a physiological assay, but considering reports that show PKCalpha activation happens on membranes (Prevostel et al., *J Cell Sci* 2000), at least the data favor our conclusion over the idea of PKCalpha phosphorylating Rab29 in the cytoplasm and then promoting lysosomal localization. This point is now clearly described in the discussion (page 10, lines 248-251).

It is not clear how the Rab29 phosphomimetics are acting as dominant active preventing lysosomal enlargement. Authors should speculate or repeat the experiments in absence of endogenous Rab29 to clarify the matter. (Reviewer #2

-- A similar concern about the effect of endogenous Rab29 was also raised by Reviewer #1 (see above). We have previously reported that knockdown of endogenous Rab29 causes the enhancement of lysosomal enlargement upon CQ treatment (Figure 5I,J of Eguchi et al, *PNAS* 2018), suggesting that the lysosome-deflating effect by phosphomimetics is a dominant active effect rather than dominant negative suppressing endogenous Rab29. This point is considered important and thus has been explained in the results section (page 7, lines 168-171).

Overall, there is some missing information regarding repeats for Western blots, such as those in figure 3C, 3D and 3E. Please add indications about repeats in the figure legend or methods. (Reviewer #2)

-- We have added the repeat information to each figure legend where it was missing. We are very sorry for the inconveniences.

The model in figure 7 however seems to suggest that Rab29 associates to lysosomal membranes independently, and is then stabilised at the membranes by LRRK2 and PKCalpha - a point which is not directly supported by the data. (Reviewer #2)

-- As noted earlier, we consider that phosphorylation of Rab29 on lysosomal membranes could retain Rab29 on the membranes for a longer time, given the present data and previous reports that phosphorylation of Rab29 is more likely to happen on the lysosomal membrane than in the cytosol. Also, as inhibition of either of the two phosphorylations ends up in disperse Rab29 localization, we have made this figure as a model of what is plausible right now. This explanation is now added in the discussion (page 10, lines 248-251).

English proofreading should be improved: "CQ was treated to HEK293" (page 4), "As we assumed that this phosphorylation is independent of LRRK2" as an opening line (page 5) (Reviewer #2)

-- Thank you for pointing out these incorrect wordings. They were corrected.

4. Description of analyses that authors prefer not to carry out

In addition, can CQ treatment drive 2xFKBP GFP-Rab29 from mitochondria to lysosomes (in the presence of rapalog and FRB-Fis1)? (Reviewer #1)

-- We do not think that a comparison between the affinities of FKBP-rapalog-FRB and Rab29-[unknown factor that directs Rab29 to lysosomes] is necessary, as the former has a Kd in the single digit nM range (Banaszynski et al, *JACS* 2005), whereas the latter (based on estimations from related PPIs) is estimated to be in the µM range, which shows a much weaker affinity than the former (McGrath et al, *Small GTPases* 2019). Furthermore, even if Rab29 appears to have migrated from mitochondria to lysosomes as a result of this experiment, one cannot rule out the possibility that a small portion of the mitochondrial membrane was incorporated into the lysosomal membrane that was enlarged by CQ treatment.

Molecular weight markes should be provided for all immunoblot experiments. (Reviewer #1)

-- The immunoblot pictures without molecular weight markers in our paper are all Phos-tag SDS-PAGE blot analyses. Phos-tag SDS-PAGE results in band shifts of phosphorylated proteins, and

© 2023. Published by The Company of Biologists under the terms of the Creative Commons Attribution License (https://creativecommons.org/licenses/by/4.0/).

writing in markers would be misleading. Moreover, previous representative studies heavily using Phos-tag (e.g., Kinoshita et al, *Proteomics* 2011, Ito et al, *Biochemical Journal* 2016) also did not show the molecular weight markers. Here we performed phos-tag SDS- PAGE analysis only to find differences in the phosphorylation state of Rab proteins.

The use of the quantification ratio of cells with Rab29-positive lysosomes in figure 1G might be slightly misleading as it does not allow the reader to understand to what extent Rab29 localisation at lysosomes upon CQ treatment. We recommend using a simpler quantification, such as by measuring the average colocalisation of Rab29 and LAMP1 per cell. (Reviewer #2) For figure 5D and 5F, As with figure 1G, we recommend using a more straightforward and impartial method of quantification such as simply measuring the colocalisation of Rab29 with LAMP1. (Reviewer #2)

-- Popular colocalization analyses using Pearson's or Mander's coefficients would be a good choice if the amounts of Rab29 varied greatly between lysosomes. However, this may not apply in this case; the amount of Rab29 or LRRK2 on each lysosome is considered to saturate quickly and a relatively low amount of them may not be detected on immunofluorescence observations, whereas the probability of finding these structures has been shown to exhibit a moderate sigmoid curve (as seen in Figure 1E or 2H of Eguchi et al., *PNAS* 2018). Therefore, the amount of Rab29 or LRRK2 could be approximated to a Bernoulli distribution in terms of colocalization with lysosomes, and this is the reason why we chose to quantify "the ratio of cells with Rab29- positive lysosomes".

We recommend using a more transparent and simple quantification method, such as average size of lysosomes per cell. (Reviewer #2)

-- As one can see in the inset of Figure 4B, unenlarged lysosomes are unfortunately too small for the quantification of their size, much less tell two small lysosomes apart in our experimental settings and laboratory resources, so we decided to analyze the largest lysosome in each cell as a representative of the cells to minimize measurement errors. This measurement only includes GFP-Rab29 positive cells, and by comparing against CQ-untreated cells we intended to increase the validity of this analysis. This quantification method was also used in our previous report (Eguchi et al, *PNAS* 2018).

First decision letter

MS ID#: JOCES/2023/261003
MS TITLE: Novel phosphorylation of Rab29 that regulates its localization and lysosomal stress response in concert with LRRK2
AUTHORS: Tomoki Kuwahara, Tadayuki Komori, Tetta Fujimoto, Maria Sakurai, and Takeshi Iwatsubo
ARTICLE TYPE: Research Article

We have now reached a decision on the above manuscript.

To see a copy of this decision letter, please go to: https://submit-jcs.biologists.org and click on the 'Manuscripts with Decisions' queue in the Author Area. (Corresponding author only has access to reviews.)

Based on your responses to the reviews obtained from Review Commons I would be happy to see a revised manuscript. We would then return it to the reviewers.

Please ensure that you clearly highlight all changes made in the revised manuscript. Please avoid using 'Tracked changes' in Word files as these are lost in PDF conversion.

I should be grateful if you would also provide a point-by-point response detailing how you have dealt with the points raised by the reviewers in the 'Response to Reviewers' box. Please attend to

© 2023. Published by The Company of Biologists under the terms of the Creative Commons Attribution License (https://creativecommons.org/licenses/by/4.0/).

all of the reviewers' comments. If you do not agree with any of their criticisms or suggestions please explain clearly why this is so.

First revision

Author response to reviewers' comments

Reviewer #1 (Evidence, reproducibility and clarity (Required)):

Summary

In this manuscript, Komori et al. examined the role of the LRRK2 substrate and regulator Rab29 in the lysosomal stress response. Briefly, in chloroquine (CQ)-treated HEK293 cells the authors observed an apparent LRRK2-independent increased in Rab29 phosphorylation which was accompanied by translocation of Rab29 to lysosomes. Intriguingly, the authors detected a similar increase in Rab29 phosphorylation when Rab29 was tethered to lysosomes in the absence of CQ treatment. Using mass spectrometry, mutagenesis and a phospho-specific anti-body, the authors mapped the CQ-induced phosphorylation site to S185 and demonstrated its independence from LRRK2. Next, the authors found that PKCa was the kinase responsible for S185 phosphorylation and lysosomal translocation of Rab29. Lastly, the authors showed that in addition to PKCa the lysosomal translocation of Rab29 was also regulated by LRRK2. Overall, Komori and colleagues provide interesting new insights into the phosphorylation-dependent regulation of Rab29. However, there are. Number of technical and conception concerns which should be addressed.
>> We thank this reviewer for taking the time to review our paper and for understanding the significance/novelty of this study. We believe we have addressed most of the concerns raised by this reviewer, as described below (some of these statements are also found in the Revision Plan submitted previously to the Review Commons).

Major points

1) Figure 1F: the localization of Rab29 to lysosomes is not convincing at all. The authors should either provide more representative image examples or image the cells at a higher resolution. The authors should also confirm the CQ-induced lysosomal localization of Rab29 in a different cell type (e.g., HEK293).
>> In Figure 1F, we have provided more representative and clearer images showing the localization of endogenous Rab29 on CQ-induced enlarged lysosomes in RAW264.7 cells. We have also added similar immunostaining images for HEK293, HeLa, A549 and MG6 (microglial) cells in Figure S2, to support the idea that this phenomenon is not cell-type specific.

Moreover, the authors should show that prenylation of Rab29 is required for its CQ-induced phosphorylation.
>> To show the requirement of prenylation for Rab29 phosphorylation, we treated cells with lovastatin, a HMG-CoA reductase inhibitor that causes the depletion of the prenylation precursor geranylgeranyl diphosphate from cells (Binnington et al, *Glycobiology* 2016, Gomez et al, *J Cell Biol* 2019), and found that lovastatin treatment almost completely blocked Rab29 phosphorylation under CQ exposure. This data is now shown in Figure S1.

2) The rapalog-induced increase in Rab29 phosphorylation in Figure 2D is not convincing since there is at least 2-3-fold more Rab29 in FRB-LAMP1 expressing cells compared to their FRB-FIS1 counterparts. An independent loading control is also missing. This is a key experiment and should be properly controlled and quantified.
>> As similar concern was also raised by Reviewer #2, we have carefully repeated the experiments, monitoring the level of alpha-tubulin as a loading control. The immunoblot images are now shown in Figure 2E and the quantification was shown in Figure 2F. Although the level of 2xFKBP-tagged Rab29 tended to increase upon forced lysosomal translocation (possibly due to unknown

© 2023. Published by The Company of Biologists under the terms of the Creative Commons Attribution License (https://creativecommons.org/licenses/by/4.0/).

stabilization mechanisms on lysosomes), the phosphorylation was detected solely upon forced lysosomal translocation, and this result was validated by calculating the percentage of phospho-Rab29 by dividing them by the sum of bands indicating non-phospho-Rab29 and phospho-Rab29.

In addition, can CQ treatment drive 2xFKBP GFP-Rab29 from mitochondria to lysosomes (in the presence of rapalog and FRB-Fis1)?
>> We do not think that a comparison between the affinities of FKBP-rapalog-FRB and Rab29-[unknown factor that directs Rab29 to lysosomes] is necessary, as the former has a Kd in the single digit nM range (Banaszynski et al, *JACS* 2005), whereas the latter (based on estimations from related PPIs) is estimated to be in the μM range, which shows a much weaker affinity than the former (McGrath et al, *Small GTPases* 2019). Furthermore, even if Rab29 appears to have migrated from mitochondria to lysosomes as a result of this experiment, one cannot rule out the possibility that a small portion of the mitochondrial membrane was incorporated into the lysosomal membrane that was enlarged by CQ treatment.

3) Figure 4A-C: Are these stable Rab29 expressing cells? If not, the quantification of "the size of largest lysosome in EACH cell" becomes very problematic. This analysis should be repeated with stable Rab29 variant cells in a background lacking endogenous Rab29.
>> The cells shown in Figure 4 are HEK293 cells transiently expressing Rab29. We are sorry that the description "the size of largest lysosome in each cell" was misleading. As we analyzed only cells overexpressing GFP-Rab29 that were marked with GFP fluorescence, we believe that transient expression should not be a problem. To avoid any misunderstandings, we have described in Figure 4 legends that only lysosomes in Rab29-positive cells (and all cells expressing Rab29) were included in the analysis of the largest lysosome of each cell.

As for the effect of endogenous Rab29 (also raised by Reviewer #2), we have previously reported that knockdown of endogenous Rab29 causes the enhancement of lysosomal enlargement upon CQ treatment (Figure 5I,J of Eguchi et al, *PNAS* 2018), suggesting that the lysosome-deflating effect by phosphomimetics is a dominant active effect rather than dominant negative suppressing endogenous Rab29. This point is considered important and thus has been explained in the results section (page 8, lines 205-208).

Furthermore, the LAMP1 signal is too dim to see any convincing colocalization (e.g., with WT) or the lack thereof (e.g., in the case of S185D).

>> We agree that the signal of LAMP1 was dim, and it turned out that the confocal microscope we used had problems with the sensitivity of the red channels. We have repeated these analyses using a new confocal microscope, and the images are now shown in new Figure 4A-B.

Lastly, the authors should corroborate their findings with an ultrastructural analysis since the electron microscopy would definitively be more suitable for this type of measurements.

>> We have obtained ultrastructural images of lysosomes in mutant Rab29-expressing cells with the help of an expert in electron microscopy (Dr. Ikuko Koyama-Honda, now included as a co-author). This time we took CLEM (correlative light and electron microscopy) images to identify the cells expressing GFP-Rab29, and the images were shown in Figure S3. As expected, most of S185D/E Rab29-expressing cells that we observed contained much smaller lysosomes as compared with WT/S185A Rab29-expressing cells, which corroborated our findings in light microcopy analysis.

4) The lysosomal colocalization of Rab29 in Figure 5C is again not convincing. This analysis needs to be repeated with high resolution imaging.

>> We have provided more representative and high-resolution images for PMA-treated RAW264.7 cells, which were taken by a new confocal microscope (the figure is now labeled Figure 5D).

Along similar lines: why not all cells in Figure 5E and Figure 5G show Rab29- and LRRK2-positive structures? How do the authors know which of these phenotypes is the prevalent one?
>> As for the ratio of cells with Rab29- and LRRK2-positive structures, it seems reasonable given that different cells have different levels of exposure to lysosomal stress and that the response is

© 2023. Published by The Company of Biologists under the terms of the Creative Commons Attribution License (https://creativecommons.org/licenses/by/4.0/).

transient and does not occur simultaneously. The ratio of these positive cells may also vary depending on the cell culture conditions. Since Rab29- and LRRK2-positive structures are rarely seen in control cells, we think this would be a meaningful phenotype even if only 20-30% of cells show such structures. The result that states that the ratio of localization changes is not 100% is now noted in the results section explaining Figure 1G (page 6, lines 136-138) where the immunocytochemical data first appears.

5) The authors need to show the level of LRRK2 depletion (Figure 6).

>> We have added the immunoblot pictures and quantification data indicating the efficient knockdown of LRRK2 protein expression in Figure S7A-B.

Given the role of LRRK2 in driving lysosomal Rab29 translocation, the importance of the LRRK2 independent pS185 for this process remains unclear.

>> Our data suggested that Rab29 is stabilized on lysosomes only when LRRK2-mediated phosphorylation and S185 phosphorylation both occur on Rab29 molecule (as shown in Figure 7 scheme), so we believe there is no contradiction. We have now described more clearly about this notion at the end of the results section (page 11, lines 284-285).

6) In general, the authors employ an alternative, biochemical assay (e.g., LysoIP) for the lysosomal translocation of Rab29. This would in particular help to clarify the effect of the Rab29 variants and LRRK2 inhibition.

>> We conducted biochemical isolation of lysosomes using dextran-coated magnetite (DexoMAG 40), as demonstrated in our previous paper (Egughi et al, *PNAS* 2018). We confirmed that the lysosomal marker protein was specifically enriched in the lysosomal fraction and that endogenous Rab29 in this fraction was much higher in CQ-treated condition (Figure 1H). We also found that the level of Rab29 in lysosomal fraction under CQ exposure decreased upon treatment with PKC inhibitor Go6983 or LRRK2 inhibitor MLi-2 (Figure S6). We believe these data would strengthen our findings derived from immunocytochemical studies.

Minor points

7) It is not clear what the authors mean by "lysosomal overload stress". Since mature lysosomal incoming pathways such as autophagy or endocytosis are disrupted by CQ, it is difficult to picture an overload. Maybe rephrasing would help to clarify this.
>> Chloroquine (CQ) is known as a lysosomotropic agent that accumulates within acidic organelles due to its cationic and amphiphilic nature, causing lysosome overload and osmotic pressure elevation, and this is what we call “lysosomal overload stress”. The well-known effects of CQ to disrupt lysosomal incoming pathways are ultimately caused by the above consequences. Also, we have previously reported that lysosomal recruitment of LRRK2 is caused by CQ but not by bafilomycin A1, the latter being an inducer of lysosomal pH elevation, or by vacuolin-1 that enlarges lysosomes without inducing lysosomal overload/pH elevation (Eguchi et al, *PNAS* 2018), and further found that not only CQ but also other lysosomotropic agents commonly induced LRRK2 recruitment (Kuwahara et al, *Neurobiol Dis* 2020). We thus have described the effect of CQ as “overload”. However, it is true that we have not provided a clear explanation for readers, so we have added some notes for lysosomal overload stress in the introduction section (page 4, lines 88-90).

8) Which cell type is used for the IF analysis in Figure 2C? This information is in general quite sparse. The authors should clearly state the cell type for each experiment/Figure.
>> We have added cell type information that was missing in several places in the manuscript. We are very sorry for the inconveniences. For clarification, HEK293 cells were used in Figure 2C.

9) Figure 2C is lacking the control IF staining for mitochondria (to which 2xFKBP-GFP-Rab29 is assumed be recruited upon co-expression with FRB-FIS1).
>> We have stained the control with mitotracker (unfortunately mitotracker Red (at 594 nm) did not work out with Alexa546 or Alexa647, so we could not obtain quadruple stains). We could see clear overlap of Rab29 with the mitotracker signal upon forced translocation, which is now shown

© 2023. Published by The Company of Biologists under the terms of the Creative Commons Attribution License (https://creativecommons.org/licenses/by/4.0/).

in Figure 2D.

10) Molecular weight markes should be provided for all immunoblot experiments.
>> The immunoblot pictures without molecular weight markers in our paper are all Phos-tag SDS-PAGE blot analyses. Phos-tag SDS-PAGE results in band shifts of phosphorylated proteins, and writing in markers would be misleading. Moreover, previous representative studies heavily using Phos-tag (e.g., Kinoshita et al, *Proteomics* 2011, Ito et al, *Biochem J* 2016) also did not show the molecular weight markers. Here we performed Phos-tag SDS-PAGE analysis only to find differences in the phosphorylation state of Rab proteins.

Reviewer #1 (Significance (Required)):

Please see above.

Reviewer #2 (Evidence, reproducibility and clarity (Required)):

The data in the manuscript convincingly demonstrates that lysosomal overload by Chloroquine treatment induces Rab29 localisation to the lysosomes and that this membrane association is dependent on PKCalpha-dependent phosphorylation at Ser185.

We have a number of rather minor comments listed below:

>> We thank this reviewer for taking the time to review our paper and for understanding the study. We have done our best to address the remaining concerns, as descried below (some of these statements are also found in the Revision Plan submitted previously to the Review Commons).

Figure 1

• Are the images in figure 1F representative? i.e. does Rab29 always colocalise to such enlarged lysosomes upon CQ treatment and does CQ treatment always drastically alter the cellular distribution of Rab29?

>> The images in Figure 1F are representative of when Rab29 is recruited, but it is not seen in all cells, and the ratio of recruitment (~80%) is shown in Figure 1G. Reviewer #1 also asked why Rab29 recruitment is not seen in all cells, and we gave the same answer above. It may be reasonable to speculate that different cells have different levels of exposure to lysosomal stress and that the response is transient and does not occur simultaneously. The ratio of these positive cells may also vary depending on the cell culture conditions. For the readers' clarity, we have added that the ratio of localization change of Rab29 is not 100% and is comparable to that of LRRK2 previously reported (page 6, lines 136-138).

• The use of the quantification ratio of cells with Rab29-positive lysosomes in figure 1G might be slightly misleading as it does not allow the reader to understand to what extent Rab29 localisation at lysosomes upon CQ treatment. We recommend using a simpler quantification, such as by measuring the average colocalisation of Rab29 and LAMP1 per cell.

>> Popular colocalization analyses using Pearson's or Mander's coefficients would be a good choice if the amounts of Rab29 varied greatly between lysosomes. However, this may not apply in this case; the amount of Rab29 or LRRK2 on each lysosome is considered to saturate quickly and a relatively low amount of them may not be detected on immunofluorescence observations, whereas the probability of finding these structures has been shown to exhibit a moderate sigmoid curve (as seen in Figure 1E or 2H of Eguchi et al., *PNAS* 2018). Therefore, the amount of Rab29 or LRRK2 could be approximated to a Bernoulli distribution in terms of colocalization with lysosomes, and this is the reason why we chose to quantify "the ratio of cells with Rab29-positive lysosomes".

Figure 2

• Considering that the "forced localisation technique" induces a non-physiological colocalization of

© 2023. Published by The Company of Biologists under the terms of the Creative Commons Attribution License (https://creativecommons.org/licenses/by/4.0/).

non-endogenous Rab29 to lysosomes, it may be an overestimation to conclude just from these data that phosphorylation of Rab29 occurs on the lysosomal surface. This is also quite in contrast with the later finding that phosphorylation by PKCalpha promotes lysosome localization of Rab29. It seems more reasonable to conclude that Rab29 can be phosphorylated when localised at the lysosomes (as opposed to other organelles such as mitochondria). If the authors feel strongly about this point they might need to find a less non-physiological assay.

>> Yes, it could be an overestimation, and as we do not have better means to conduct a less non-physiological assay, we have modified the description from "occurred on the lysosomal surface" to "could occur on the lysosomal surface" (page 6 line 143 (subtitle), page 7 line 160, and the title of Figure 2).

Regarding the comparison with the later finding that phosphorylation by PKC promotes lysosome localization of Rab29, these data (Figure 2 and 5) could be explained with a single speculation: phosphorylation of Rab29 on lysosomal membranes could retain Rab29 on the membranes for a longer time. It is not easy to decipher which comes first, association with membranes or phosphorylation of Rab29, in a physiological assay, but considering reports that show PKC activation happens on membranes (Prevostel et al., *J Cell Sci* 2000), at least the data favor our conclusion over the idea of PKCs phosphorylating Rab29 in the cytoplasm and then promoting lysosomal localization. This point was already mentioned in the result section (page 10, lines 262-265) but is now additionally described in the discussion (page 12, lines 298-304).

• The increasing levels of non-phosphorylated Rab29 over the indicated time course of AP21967 treatment in Figure 2B are concerning. First, could you provide an explanation for this clear increase in both non-p-Rab29 and p-Rab29 in the phostag but not the normal gel? Second, could all quantifications of p-Rab29 be made relative to the non-p-Rab29?

>> As similar concern was also raised by Reviewer #1, we have repeated the experiments and made quantifications, monitoring the level of alpha-tubulin as a loading control. The immunoblot images are now shown in Figure 2E and the quantification was shown in Figure 2F. Although the level of 2xFKBP-tagged Rab29 tended to increase upon forced lysosomal translocation (possibly due to unknown stabilization mechanism on lysosomes), the phosphorylation was detected solely upon forced lysosomal translocation, and this result was validated by calculating the percentage of phospho-Rab29 by dividing them by the sum of bands indicating non-phospho-Rab29 and phospho-Rab29.

Figure 3

• It is not clear how the Rab29 phosphomimetics are acting as dominant active preventing lysosomal enlargement. Authors should speculate or repeat the experiments in absence of endogenous Rab29 to clarify the matter.

>> A similar concern about the effect of endogenous Rab29 was also raised by Reviewer #1. We have previously reported that knockdown of endogenous Rab29 causes the enhancement of lysosomal enlargement upon CQ treatment (Figure 5I,J of Eguchi et al, *PNAS* 2018), suggesting that the lysosome-deflating effect by phosphomimetics is a dominant active effect rather than dominant negative suppressing endogenous Rab29. This point is considered important and thus has been explained in the results section (page 8, lines 205-208).

• Overall, there is some missing information regarding repeats for Western blots, such as those in figure 3C, 3D and 3E. Please add indications about repeats in the figure legend or methods.

>> We have added the information about the number of experimental repeats, such as the description "Representative image of n = XX trials", in the legends of Figure 1A, 1B, 1D, 1H, 2B, 2E, 3C, 3D, 3E, 5C, S1A, S4A, S5B, S5C, S6A and S7.

Figure 4

• We recommend using a more transparent and simple quantification method, such as average size

© 2023. Published by The Company of Biologists under the terms of the Creative Commons Attribution License (https://creativecommons.org/licenses/by/4.0/).

of lysosomes per cell.
>> As one can see the pictures in Figure 4B, unenlarged lysosomes are unfortunately too small for the quantification of their size, much less tell two small lysosomes apart in our experimental settings and laboratory resources, so we decided to analyze the largest lysosome in each cell as a representative of the cells to minimize measurement errors. This measurement only includes GFP-Rab29 positive cells, and by comparing against CQ-untreated cells we intended to increase the validity of this analysis. This quantification method was also used in our previous report (Eguchi et al, *PNAS* 2018).

Figure 5

• To further demonstrate that PKCalpha phosphorylates endogenous Rab29 at Ser185, we recommend reperforming the Go3983/PMA treatment in figure B with the anti-p-Ser185 antibody. It may be sufficient to perform the treatment only at 4 or 8 hours, simply to provide stronger evidence regarding the phosphorylation of endogenous Rab29.
• It is not clear whether the activity of PMA in the assay is due to inhibition of PKCalpha. Are the effects ablated by PKCalpha KD

>> To simultaneously address these two issues, we knocked down PKCα, β, γ and δ, known PKC isoforms targeted by both PMA and Go6983, followed by PMA treatment and examined the changes of Ser185 phosphorylation by immunoblotting. As shown in new Figure 5C, PMA treatment enhanced Ser185 phosphorylation, whereas this effect was not suppressed by knockdown of either of the PKC isoforms, including PKCα. However, since knockdown of PKCα or PKCδ showed a slight tendency to suppress phosphorylation, we knocked down both at the same time and could see the suppression of phosphorylation (Figure 5C top and middle panels). We also found that phosphorylations of other substrates of PKC were largely suppressed by simultaneous knockdown of PKCα and PKCδ (Figure 5C bottom panel), suggesting that these two isoforms act dominantly and compensatory in our cells. Therefore, it would also be possible that other PKCs may work in other types of cells where that isoforms are more active. These results and notions are now described in the results (page 10, lines 246-254) and discussion (page 13, lines 333-338).

We also tried to confirm whether PKCδ directly phosphorylates Rab29 in vitro, but unlike PKCα, no clear phosphorylation was observed. The difference from the results observed in cells is unknown, but in vitro environment is more artificial, and even if in vitro results were as expected, it is still unclear whether PKCs really directly phosphorylate Ser185 in cells. So we decided to show only the results in cells and instead add these notions in the discussion (page 13, lines 338-343) with the statement that "the possibility remains that PKCs phosphorylate Rab29 both directly and indirectly in cells". We also changed the subtitle in the text (page 9, line 226) and the title of Figure 5, from "PKCalpha phosphorylates Rab29 at Ser185 and regulates lysosomal localization of Rab29" to "PKCs are involved in Ser185 phosphorylation and lysosomal localization of Rab29".

• For figure 5D and 5F, As with figure 1G, we recommend using a more straightforward and impartial method of quantification such as simply measuring the colocalisation of Rab29 with LAMP1.

Figure 6

• Again, we recommend altering the methods of quantification

>> The answer to these concerns is the same as above for Figure 1G; we think that linear colocalization analyses are not suitable in this case because it would not reflect the dynamics of Rab29.

Figure 7

• The model in figure 7 however seems to suggest that Rab29 associates to lysosomal membranes independently, and is then stabilised at the membranes by LRRK2 and PKCalpha - a point which is not directly supported by the data.

© 2023. Published by The Company of Biologists under the terms of the Creative Commons Attribution License (https://creativecommons.org/licenses/by/4.0/).

>> As mentioned above for Figure 2, we consider that phosphorylation of Rab29 on lysosomal membranes could retain Rab29 on the membranes for a longer time, given our present data on lysosomal force-localization of Rab29 (Figure 2B, E) as well as previous reports that both PKCs and LRRK2 are known to be active on membranes (Gomez et al., *J. Cell Biol*. 2019, Prevostel et al., *J. Cell Sci*. 2000) and that phosphorylation of Rab29 is more likely to happen on the membranes than in the cytosol (Liu et al., *Hum. Mol. Genet*. 2017). This explanation is now described in the discussion (page 12, lines 298-304) in addition to that in the result section (page 10, lines 262-265). Also, as inhibition of either of the two phosphorylations ends up in disperse Rab29 localization, we have made this figure as a model of what is plausible right now.

English proofreading should be improved: "CQ was treated to HEK293" (page 4), "As we assumed that this phosphorylation is independent of LRRK2" as an opening line (page 5),

>> Thank you for pointing out these incorrect wordings. They were corrected.

Reviewer #2 (Significance (Required)):
These cell biology findings are important in the field as both Rab29 and LRRK2 are implicated in the pathogenesis of Parkinson disease. The phosphorilation of Ser185 of Rab29 by PKCalpha is novel and contributes to our understanding of Rab29 and LKRR2 regulation. One limitation of the study is that is conducted in only two cell types quite unrelated to the disease, so how general and disease relevant are the findings it is not clear. Most of the data are solid. There are two experiments whose results are difficult to interpret and a few controls missing. Also a few issues with quantifications, all of which is described in details above and will need to be fixed prior to publication. My expertise for this paper is in the cell biology of lysosomal function.

>> The issue that only two cell types were analyzed was also raised by Reviewer #1 (major point 1 above), we have examined additional cell types including HEK293, HeLa, A549 and MG6 (microglial) cells for endogenous Rab29 relocalization to enlarged lysosomes. These data are now shown in Figure S2. Our responses to other issues raised are described elsewhere. Thank you for these insightful comments.

Other changes

- Following the instruction of *J. Cell Sci.*, Changes made in the revised manuscript were highlighted in blue. Bar graphs were changed to plotted graphs to visualize the spread of individual values. Uncropped images of all immunoblots shown in the figures were provided in Fig. S8, with the name “Blot transparency”.

- Immunoblot images previously shown in Fig. S1A (now in Fig. S4A) were replaced with better ones that had been taken in another round of experiment.

- Author list: we have now invited Dr. Ikuko Koyama-Honda (performed CLEM experiments) and Dr. Mitsunori Fukuda (provided materials and supervised the study) as co-authors. All authors approved the addition of these co-authors.

Second decision letter

MS ID#: JOCES/2023/261003
MS TITLE: Novel phosphorylation of Rab29 that regulates its localization and lysosomal stress response in concert with LRRK2

AUTHORS: Tadayuki Komori, Tomoki Kuwahara, Tetta Fujimoto, Maria Sakurai, Ikuko Koyama-Honda, Mitsunori Fukuda, and Takeshi Iwatsubo

ARTICLE TYPE: Research Article

© 2023. Published by The Company of Biologists under the terms of the Creative Commons Attribution License (https://creativecommons.org/licenses/by/4.0/).

Thank you for sending your manuscript to Journal of Cell Science through Review Commons.

I am happy to tell you that your manuscript has been accepted for publication in Journal of Cell Science, pending standard ethics checks. Where referee reports on this version are available, they are appended below.

© 2023. Published by The Company of Biologists under the terms of the Creative Commons Attribution License (https://creativecommons.org/licenses/by/4.0/).
